# Characterization of Sequence Distributions in Random and Semi-Random Copolymers

**DOI:** 10.1021/acs.macromol.5c01799

**Published:** 2026-03-03

**Authors:** Michael Cole, Jordan Fitch, Tara Y. Meyer

**Affiliations:** Deparment of Chemistry, 6614 University of Pittsburgh, Pittsburgh, Pennsylvania 15260 United States

## Abstract

We report a strategy for analyzing and distinguishing the sequence distributions of random and semirandom poly­(lactic-*co*-glycolic acid) (PLGA) analogs using selective digestion at cleavable olefin-containing monomer units. Semirandom copolymers were synthesized via a parallel-successive (P-S) approach that enables coarse-grained sequence control by coupling telechelic oligomers of varied composition and length. Following cross-metathesis digestion, the resulting fragment distributions were fractionated and analyzed via NMR, SEC, and MALDI-MS. These postdigestion data directly reflect the microstructural arrangement of the cleavable units in the predigestion copolymers. Monte Carlo simulations were employed to model both random and P-S copolymerizations, offering in silico digestion data that elucidate the influence of oligomer feed ratios and dispersity on the resulting block-length distributions. Experimental and simulated results demonstrate that P-S copolymers exhibit broader and sometimes bimodal fragment distributions compared to their random analogs, validating the method’s capacity to encode and detect distinct microstructural features. This approach provides a scalable, analytically tractable platform for tuning and characterizing sequence distributions in degradable polyesters and potentially other polymer systems where sequence plays a critical role in material properties.

## Graphical abstract

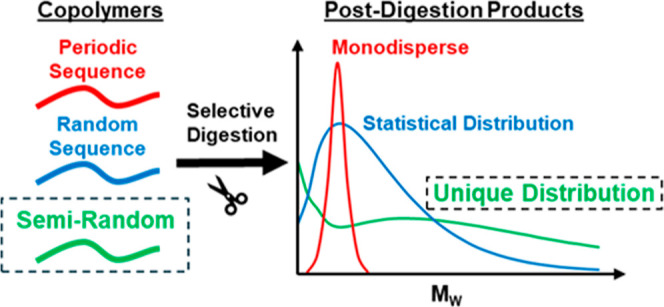

## Introduction

Herein, we demonstrate how selective digestion can serve as a powerful tool for characterizing and distinguishing the microstructures of semirandom polymers, which are notoriously difficult to analyze. We first synthesize semirandom and random copolymers that include a cleavable unit (Figure 1). After subjecting these copolymers to selective digestion and examining the molecular weights of the resulting fragments, the sequence distributions are compared and shown to reflect the semirandom characteristics of the microstructures. The experiments are further supported by in-silico digestion data generated from Monte Carlo simulations.

**Figure 1:**
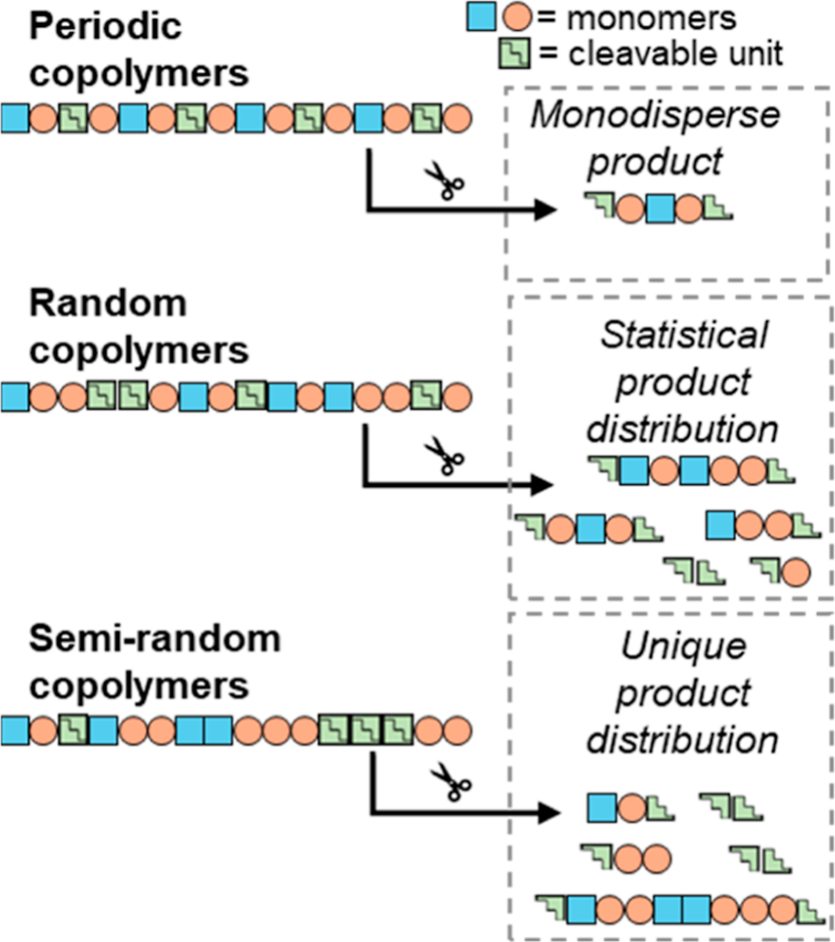
Effects of polymer microstructure on the products of polymer digestion at an embedded cleavable unit.

We were motivated to develop this degradation-based strategy by our interest in polymers bearing lactic/glycolic acid units that exhibit sequence-tunable degradation properties. Compared to the synthesis of perfectly periodic copolymers, preparing semirandom polymers offers a more scalable and practical alternative. We are currently exploring an approach to synthesize semirandom copolymers. This strategy, termed parallel-successive (P-S) copolymerization, involves synthesizing two distinct sets of chemically reactive telechelic oligomers. Upon coupling, these oligomers form semirandom polymers with coarse-grained sequence control and tunable degradation properties. These features can be tailored by adjusting the oligomer compositions prior to polymerization. This synthetic scheme should be adaptable to flow-based processing^1^ offering a promising route for large-scale production. While the P-S approach is well-established in polyurethane chemistry^2^ the application of this method to control sequence distributions in other materials is rare.

Although changing the P-S conditions will systematically change the microstructure of the resulting polymers, the characterization and differentiation of these new materials presents unique challenges that we believe can be addressed in part by selective digestion. To investigate both the synthesis and the subsequent characterization of the polymer microstructures produced by a P-S methodology, we are focusing on an AA/BB step-growth system where one AA monomer is terminated with the alcohols from glycolic acid and two BB monomers are terminated with carboxylic acids from lactic acid (Figure 2). One of the BB monomers features a cleavable olefin, which can be selectively digested. The molecular weight distributions (MWDs) of the postdigestion fragments should reflect the distribution of alkenes in the chain. An AA/BB assembly (rather than AB) has been used because this approach allows for stoichiometric control of oligomer molecular weight distributions and also creates monomer pools with well-defined end groups.

**Figure 2:**
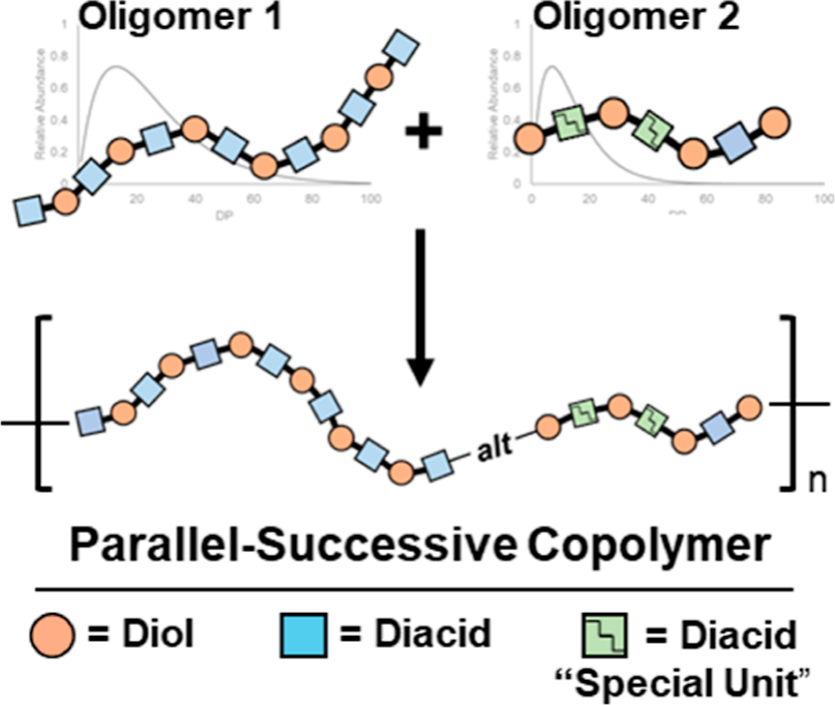
Parallel-successive copolymerization: two sets of oligomers are synthesized in parallel before being coupled in a separate polymerization step. In the current work, the “Special Unit” is a metathesis-cleavable alkene.

Monomer sequence control offers a relatively unexplored route to tuning polymer bulk properties.^3–5^ Examples of sequence-sensitive and sequence-tolerant polymer properties exist in both biological^6,7^ and synthetic systems.^8–10^ The degree to which monomer sequence affects polymer bulk properties, however, depends on the specific chemical system and the mechanism through which that property manifests itself.^11–13^ In DNA, for example, a single monomer “error” can drastically change its function, whereas protein function often depends more on higher-order folding, and many sequence variations can be tolerated without significantly affecting performance. Sequence effects also play important roles in synthetic systems. For example, conjugated oligomers show sequence-dependent optoelectronic properties^14^ and α-hydroxy acid-derived polyesters have degradation profiles that vary with monomer sequence.^5,8^

Our group has previously shown that certain fully sequenced poly­(lactic-*co*-glycolic acid) (PLGA) copolymers degrade more slowly than conventional random PLGA. For instance, poly­(lactic-*alt*-glycolic acid) (poly­(LG)), which has no glycolic acid–glycolic acid (G–G) linkages, undergoes predominantly end-chain scission, resulting in slower degradation.^15^ In contrast, random PLGA contains glycolic acid–rich segments and degrades more quickly through intrachain scission at G–G sites. We have further demonstrated that introducing glycolic acid “errors” into poly­(LG) progressively accelerates its degradation.^11,16^ These observations point to a high level of sequence-based tunability in polyester systems such as PLGA, potentially broadening their application space.

The choice of synthesis methodology and monomer chemistry strongly influences the degree and type of sequence control that can be achieved. Broadly, sequence control can be approached from either a top-down or bottom-up perspective (Figure 3). Top-down approaches typically begin with a random, one-pot polymerization. For example, reversible deactivation radical polymerization methods accomplish sequence control either through reactivity ratios or time-based monomer additions.^17,18^ In contrast, bottom-up strategies allow for more intricate and precisely defined sequence patterns. These results can be achieved through a variety of methods including single-unit monomer insertion (SUMI),^19^ iterative exponential growth (IEG),^20^ and solid-phase synthesis.^21^ A bottom-up method used extensively by our group is segmer assembly polymerization, wherein discrete sequenced macromonomer building blocks (segmers) are assembled to give periodic microstructures.^22^

**Figure 3:**
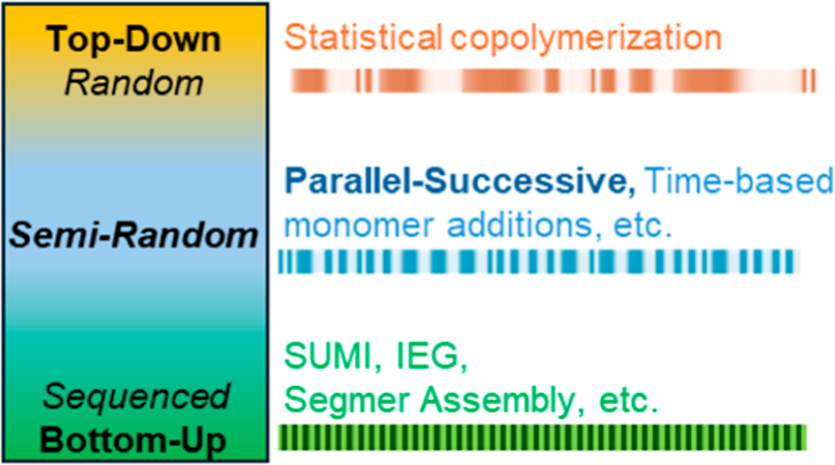
Depiction of top-down versus bottom-up sequence control; SUMI = single-unit monomer insertion, IEG = iterative exponential growth.

Sequenced copolymers synthesized via random and statistical methods have the advantage of being comparatively simple to set up and are more easily scaled up relative to bottom-up approaches but are typically limited in the possible sequence patterns given a defined set of monomer building blocks. The parallel-successive copolymerization method has the potential to bridge this gap, exploiting the relative simplicity of statistical copolymerizations while broadening their accessible sequence space.

The model system used in the current work is designed to demonstrate that our P-S approach leads to polymer sequence distributions that are predictable and differentiable from what we would expect if the monomers were simply mixed in a one-pot reaction. With this tool in hand, we can then consider how to exploit this approach for a variety of applications related to PLGA analogs and other step-growth systems.

### Sequence Characterization

The analytical techniques that are widely used to characterize polymer sequences,^23^ such as NMR spectroscopy and mass spectrometry, have limitations for polymers that are neither fully sequenced nor purely random.^24^ ^1^H and ^13^C NMR, for example, provide high-resolution sequence information if the monomers are relatively small, e.g., propylene and lactic acid. Larger or more complex monomers, where the monomer variation is not proximate to the backbone, do not yield significant sequence based differences in the NMR spectra.^25^

Mass spectrometry (MS) and MS–MS fragmentation patterns have also been used to determine sequence distribution information in sequenced polymers^26,27^ Techniques like matrix-assisted laser-desorption ionization (MALDI) MS can reveal overall polymer composition, while MALDI-MS-MS can provide sequence-specific fragmentation patterns.^28–31^ For example, Lutz and co-workers have used MS–MS secondary fragmentation to identify the sequence of fully sequenced polymers with precise molecular weights.^30^ However, quantifying polymer sequences via MS or MS–MS is not trivial: low-molecular-weight bias in ionization and the complexity of fragmentation spectra often interfere with interpretation.^32^ Also, in MS–MS studies on PLGA and its analogs conducted by our laboratory, the abundance of cleavable ester groups leads to numerous fragmentation paths, which inevitably produces fragments with different sequences but the same mass. Notably, the Lutz system was designed to avoid these issues.

Although nonselective MS fragmentation does not yield useful sequence information in our case, the introduction of a selective cleavage method is potentially powerful. We propose to characterize our P-S copolymers using selective chemical degradation followed by analysis of the fragments. This approach has been used extensively for biological polymers. For example, restriction digestion, where enzymes cleave DNA chains at specific sites, is a method for analyzing DNA sequences; the DNA fragments can then be amplified, separated, and analyzed.^33^ This process of digestion, separation and characterization is also applicable to synthetic polymer systems that possess specific cleavable groups. In fact, many examples of depolymerization exist in the literature, including the depolymerization of polysaccharides via ozonolysis^34^ and polyolefins via cross-metathesis.^35,36^ Depolymerization has also been used specifically as a tool for sequence determination as far back as 1939, where the microstructure of butadiene copolymers was studied after subjecting the copolymers to ozonolysis.^37,38^ Synthetic work has also been done to determine the hard-block length distributions of polyurethanes by synthesizing polyester-polyurethane copolymers, subjecting them to partial hydrolytic degradation, and analyzing the molecular weight distributions of the resulting fragments.^39^

Another major tool for analyzing the microstructure of random and semirandom copolymers, which we have applied and use to support our degradation analysis, is Monte Carlo simulations. By creating model polymers using the same monomer feeds and reaction order, we can approximate the likely sequence profiles. Previous computational models for related polyurethane systems have served as inspiration. In canonical work from the 1970s, Peebles mathematically demonstrated that “hard-block segments” in polyurethanes generally follow the most probable statistical distribution.^40,41^ Later, Speckhard et al. expanded upon these ideas to simulate compositions and molecular weight distributions by manipulating the average degree of polymerization and relative reaction kinetics.^42^ Alternative computational approaches, including the application of machine learning to pyrolysis mass spectrometry, have demonstrated significant potential for sequence analysis.^43^

## Results

### Synthesis Overview

All monomers were diacids or diols terminated by *l* -lactic acid or glycolic acid, and were synthesized using a series of orthogonal protections and deprotections followed by ester-forming Steglich esterifications as previously reported by our group.^25^ All acids were protected with benzyl (Bn) groups and alcohols with *tert*-butyldiphenylsilyl (Si) groups. All ester couplings were accomplished using dicyclohexylcarbodiimide (DCC) or diisopropylcarbodiimide (DIC) as the coupling reagent and 4-(*N*,*N*-dimethylamino)­pyridinium 4-tolunesulfonate (DPTS) as the catalyst. Detailed experimental information for the monomer and polymer syntheses are provided in the Supporting Information.

### Synthesis of Monomers

The glycolic acid-derived symmetric diol (**G**) was synthesized by coupling the free acid of TBDPS protected glycolic acid to a symmetric diol-terminated alkyl linker to form the palindromic silyl protected diol, which was subsequently deprotected using tetrabutylammonium fluoride (TBAF) to form the diol **G** (Scheme 1).

**Scheme 1:**
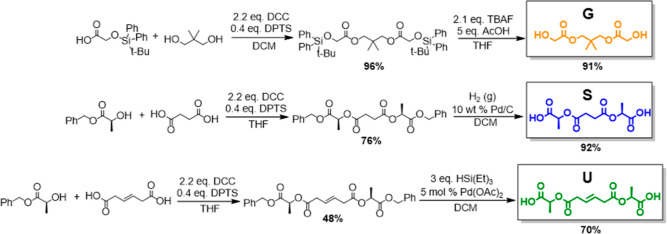
Synthesis of **G**, **S** and **U** Monomers

Similarly, two lactic acid-terminated symmetric diacids were synthesized by coupling the free alcohols of Bn-protected *l* -lactic acid with a symmetric acid-terminated alkane linker and a symmetric acid-terminated linker containing an alkene, respectively, to form the two palindromic benzyl diesters. The esters were deprotected by selective palladium-catalyzed hydrogenolysis to form the two diacids, one with an unsaturated alkene linker (**U**) and one with a saturated linker (**S**).

### Random Copolymer Synthesis

To prepare random copolymers of **G** with **U** and/or **S**, the monomers were combined in the appropriate specified ratios. After the addition of the DPTS catalyst and DIC coupling reagent, the mixtures were stirred for 3 h. The polymers were precipitated in methanol (Scheme 2).

**Scheme 2:**
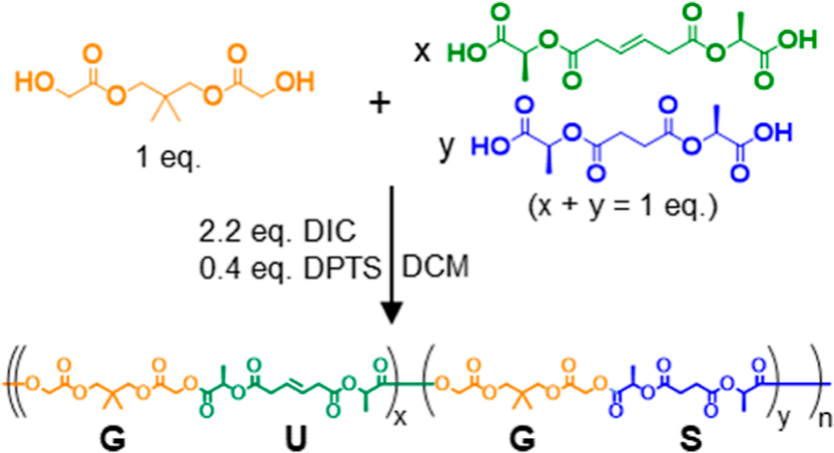
Example Synthesis of a Random Copolymer Including **G, U** and **S**

### Parallel-Successive (P-S) Copolymer Synthesis

The P-S copolymers were synthesized by first preparing two sets of oligomers whose molecular weight averages were determined by stoichiometric imbalance. In the first oligomerization reaction, excess **U**/**S** diacid, DPTS and DIC were combined, followed by the slow addition of the **G**. In parallel, the second oligomerization reaction was set up by combining excess **G** diol, DPTS and DIC, followed by the slow addition of the **S** and/or **U** diacid mixture. The exact monomer ratios for each oligomerization reaction depended upon the desired oligomer DP and were calculated using Carothers’ equation under the conditions of stoichiometric imbalance, where the reaction has gone to completion (eq 1). Under these conditions, Carothers’ equation simplifies to

1 $$\overset{®}{DP}=\frac{1+r}{1-r}$$

where “*r*” is the stoichiometric imbalance ratio and “ $\overset{®}{DP}$ ” is the expected average degree of polymerization.

These oligomerizations were allowed to proceed for 3 h before being combined. After 12 h, the P-S copolymers were collected by precipitation in methanol (Scheme 3). Experimentation revealed that the **U** monomer incorporation into short-chain diacid oligomers required a higher loading than theoretically predicted by the Carothers equation to achieve the target P-S copolymer composition. The most probable cause for this observation is the generation of *N*-acylurea biproducts from the reaction between excess carbodiimide and the free carboxylic acid end groups of the capped oligomers after the diol monomer was fully consumed. This side-reaction consumes some diacid end groups, making them unavailable for the polymerization step and slightly reducing the actual average DP of the chains. Increased **U** loading in the P-S copolymer was required to achieve a comparable composition profile to its random analog by NMR.

**Scheme 3:**
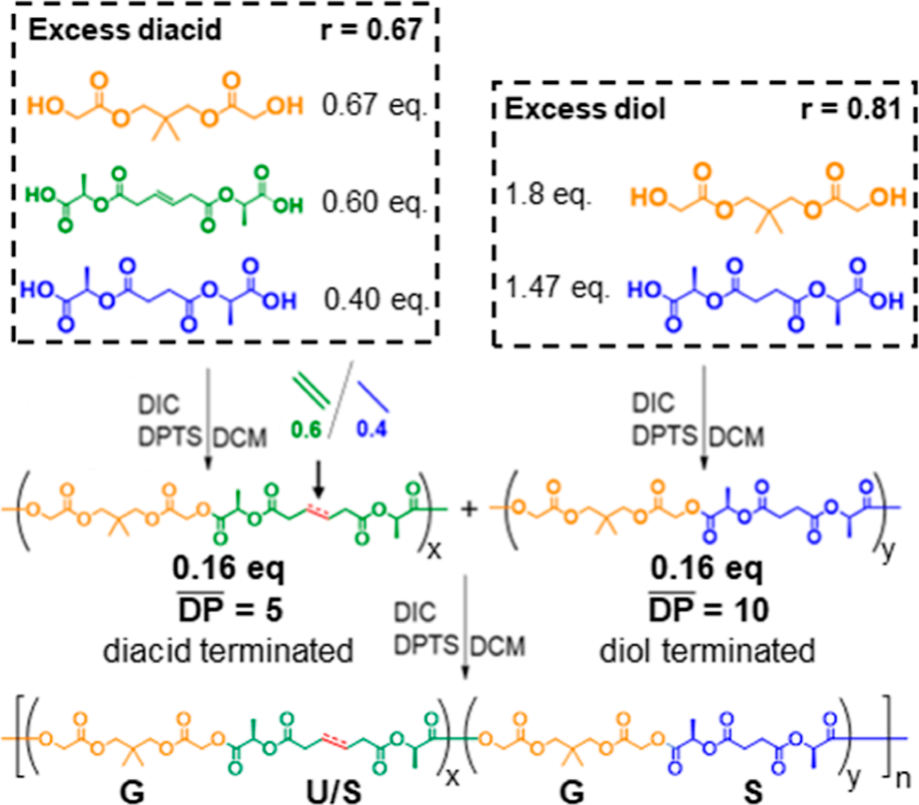
Example Synthesis of the P-S Copolymer, **(U60)**_**5**_ **-** *alt* **-(U0)**_**10**_ ^a^

a The calculated excess monomer in each oligomer reaction limits the molecular weights in a controlled manner and caps each oligomer chain with identical end groups, to enable a final AA/BB final polymerization reaction. For simplicity the end groups are not depicted for the intermediate macromonomers. The diacid terminated oligomer (left) contains a 60/40 mixture of **U** and **S** respectively. The average DPs (5 or 10) represent the number of monomers per chain (**G** and **U**/**S**). The overall combined amount of diol (**G**) and diacid (**U**/**S**) monomers between the two oligomerization reactions is stoichiometrically balanced

### Polymer Nomenclature

The polymers in this study have an alternating structure, with **G** units between **S** or **U** units. **S** and **U** are distinguished by the presence of an unsaturated olefin linker embedded within **U**, which allows the polymers to be digested via cross-metathesis after the polymerization is complete. Any compositional variation between polymers in this study is due to alterations in their **U**:**S** ratio. Random copolymers will therefore be named according to their **U** content. For example, **Rand­(U20)** refers to the random copolymer **poly­(G-** *alt* **-(U**_**0.2**_ **S**_**0.8**_ **))**, where 20% of the diacid units contain an unsaturated carbon–carbon bond and **Rand­(U0)** refers to **poly­(G-** *alt* **-S)** that contains no unsaturated units. P–S copolymers will be identified by the feed ratio of **U** monomer and degree of polymerization (DP) of each oligomer stream prior to coupling. For example, a P–S copolymer synthesized from oligomers with average DPs of 5 and 10 and **U** feed ratios of 60% and 0%, respectively, would be named **(U60)**_**5**_ **-** *alt* **-(U0)**_**10**_. Using this system, a P–S copolymer synthesized from oligomers with an average DP of 5 and 10 and **U** content of 0% and 30%, respectively, would be named **(U0)**_**5**_ **-** *alt* **-(U30)**_**10**_.

### Polymer Digestion

To allow for analytical digestion that would lead to information about the sequence distribution, alkene-containing linkers were embedded in the polymer through incorporation of varying amounts of the **U** monomer. To study the distribution of the **U**-derived units in the polymer, the olefin-containing units were cleaved using cross-metathesis and the molecular weight distributions of the resulting fragments were determined by size-exclusion chromatography (SEC). Digestions were carried out by combining the polymer, excess styrene, and Grubb’s second generation catalyst (G2) in 1,2-dichloroethane at 50 °C for approximately 24 h. The styrene was added to the reaction mixture to improve the uniformity of the end groups after cleavage, facilitating MS analysis. Detailed information about all metathesis digestion conditions can be found in the Supporting Information (Figures S19 and S20). A typical reaction scheme for the digestion of **Rand­(U20)** with relevant characterization data is shown in Figure 4. Molecular weight and composition data for all digested polymers are summarized in Table 1.

**Figure 4:**
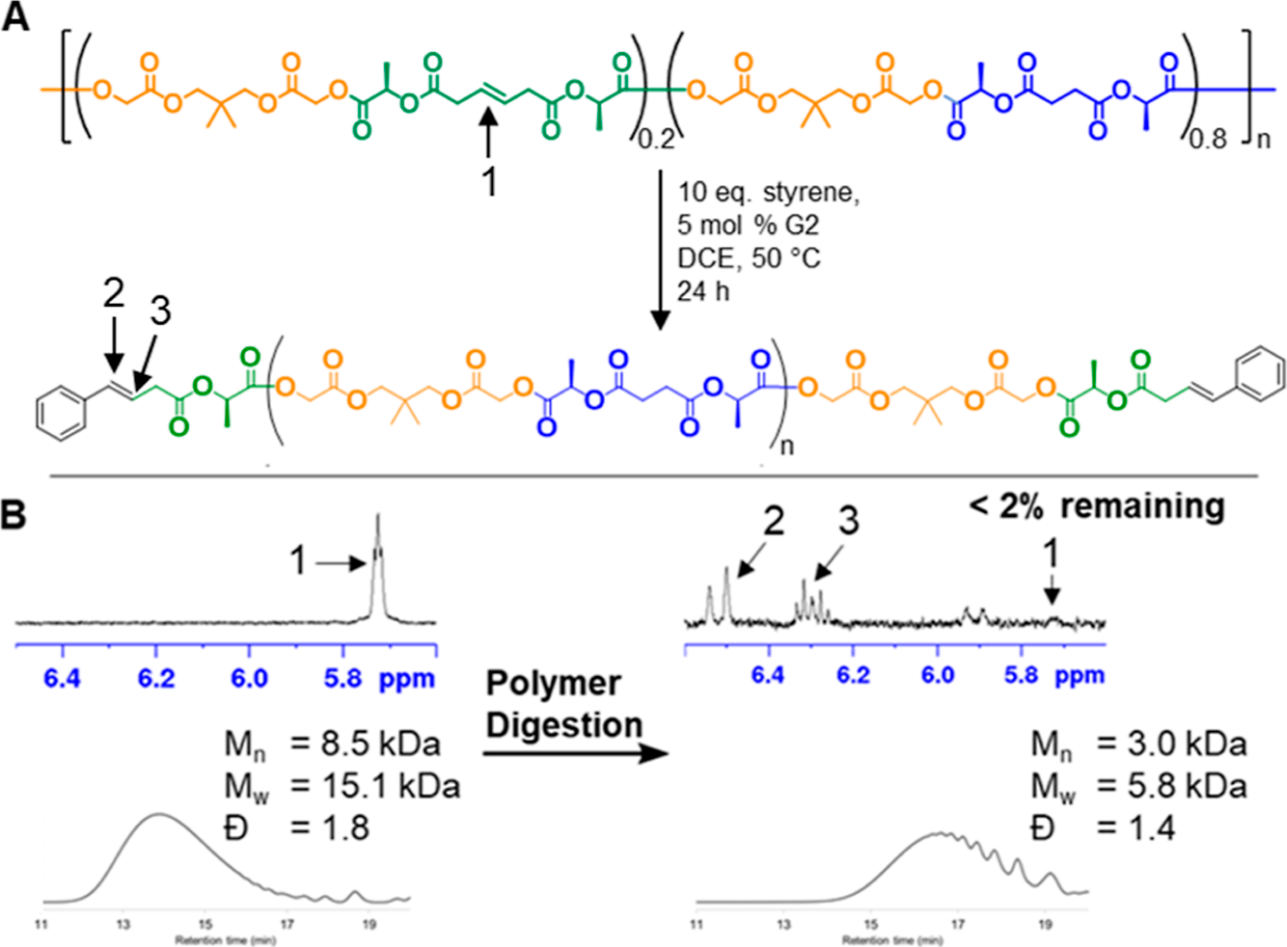
(A) Typical polymer digestion conditions for **Rand­(U20)**; (B) ^1^H NMR (top) and SEC (bottom) data of the copolymer **Rand­(U20)** before and after digestion.

**Table 1:** Polymer Molecular Weight and Composition Data^a^ ^,^ ^c^

polymer name	% U^b^	*M*_n_ ^b^ (kDa)	Đ^b^
**Rand(U80)**	74.1	9.6	1.4
**Rand(U80)D**	<2	2.0	1.2
**Rand(U20)** trial 1	18.6	8.5	1.8
**Rand(U20)D trial 1**	<2	3.0	1.4
**Rand(U20)** trial 2	17.9	9.2	1.4
**Rand(U20)D trial 2**	<1	4.0	1.6
**(U60)**_**5**_ **-** *alt* **-(U0)**_**10**_	19.0	7.8	1.4
**(U60)**_**5**_ **-** *alt* **-(U0)**_**10**_ **D**	<1	4.0	1.8

a Determined by ^1^H NMR.

b Determined by SEC equipped with two columns (TSKgel-G3000H, TSKgel-G4000H) equipped with RI detector calibrated vs polystyrene standards. %U is defined as the molar percentage (100*U/(U + S)).

c “D” suffix appended to sample name after digestion.

### Polymer Fractionation and Characterization

Taking inspiration from Hawker and co-workers,^44^ fractionation of the digested polymer samples was performed to aid in their analysis. An SEC equipped with an automated fractionator was used to perform analytical scale fractionation of postdigestion polymer samples to facilitate characterization. All samples were autofractionated using the same time-based method. Fractionation greatly improved the MALDI spectra because the narrower dispersity of the individual fractions increases the proportion of higher molecular weight species that are successfully ionized overall. SEC traces for the fractionated postdigestion **Rand­(U20)­D trial 1** samples are shown in Figure 5 and the MALDI data for each of these fractions are shown in Figure 6. The SEC data for the fractionated polymers are shown in Figure 7.

**Figure 5:**
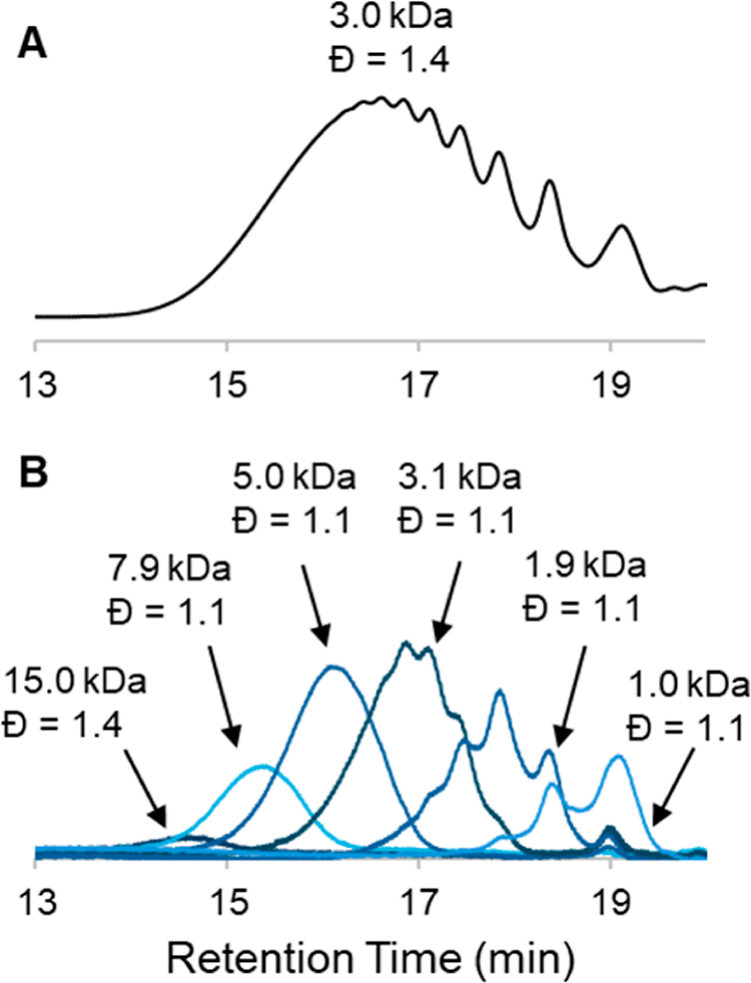
SEC data of digested **Rand­(U20) Trial 1** before (A) and after (B) fractionation.

**Figure 6:**
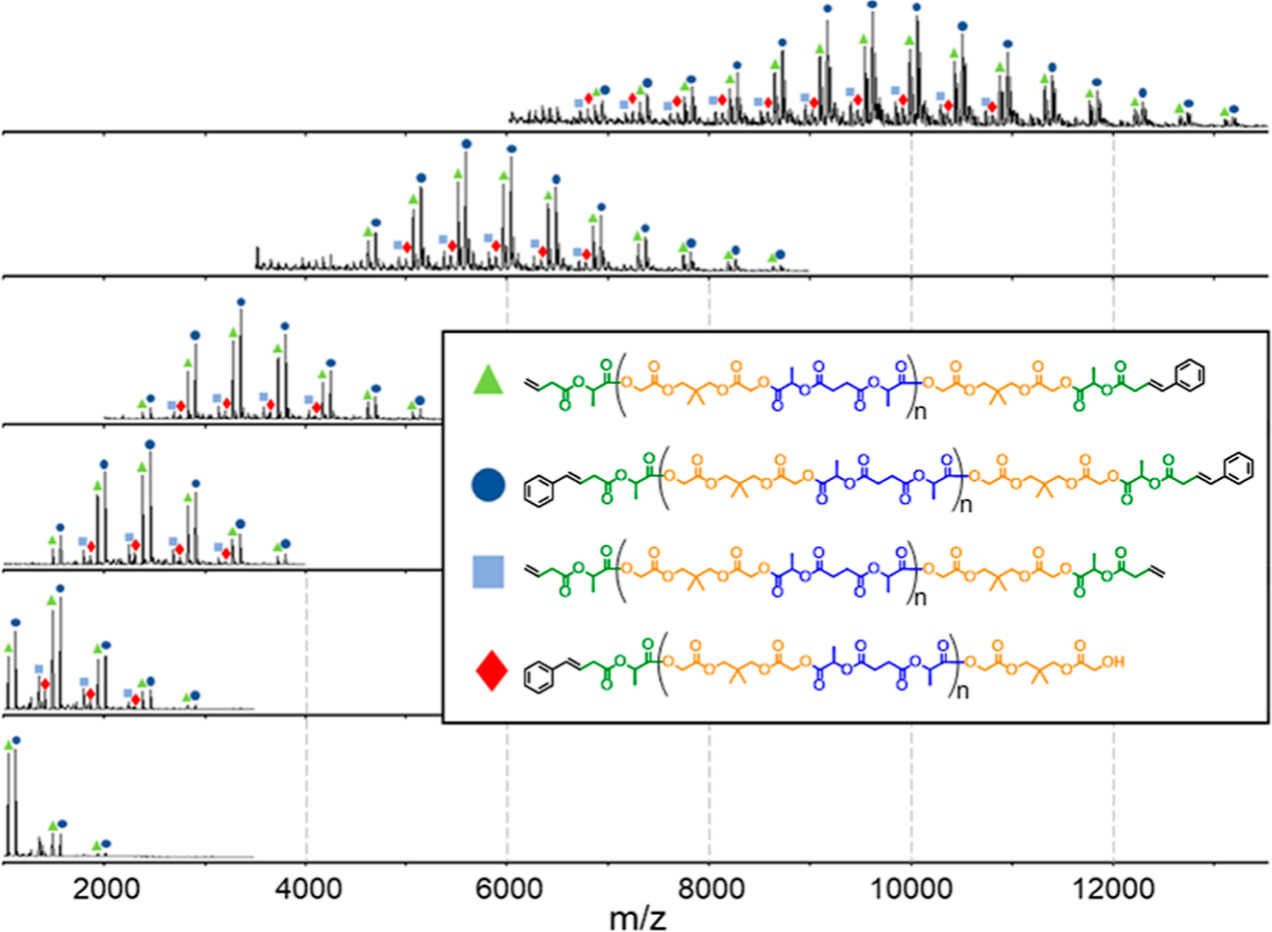
Positive-mode MALDI spectra for digested **Rand­(U20) Trial 1** after fractionation. The four major end group populations are shown. Phenyl-terminated chains were generated by cross metathesis with styrene that was added during digestion. Ethylene produced during metathesis led to terminal olefin end group formation.

**Figure 7:**
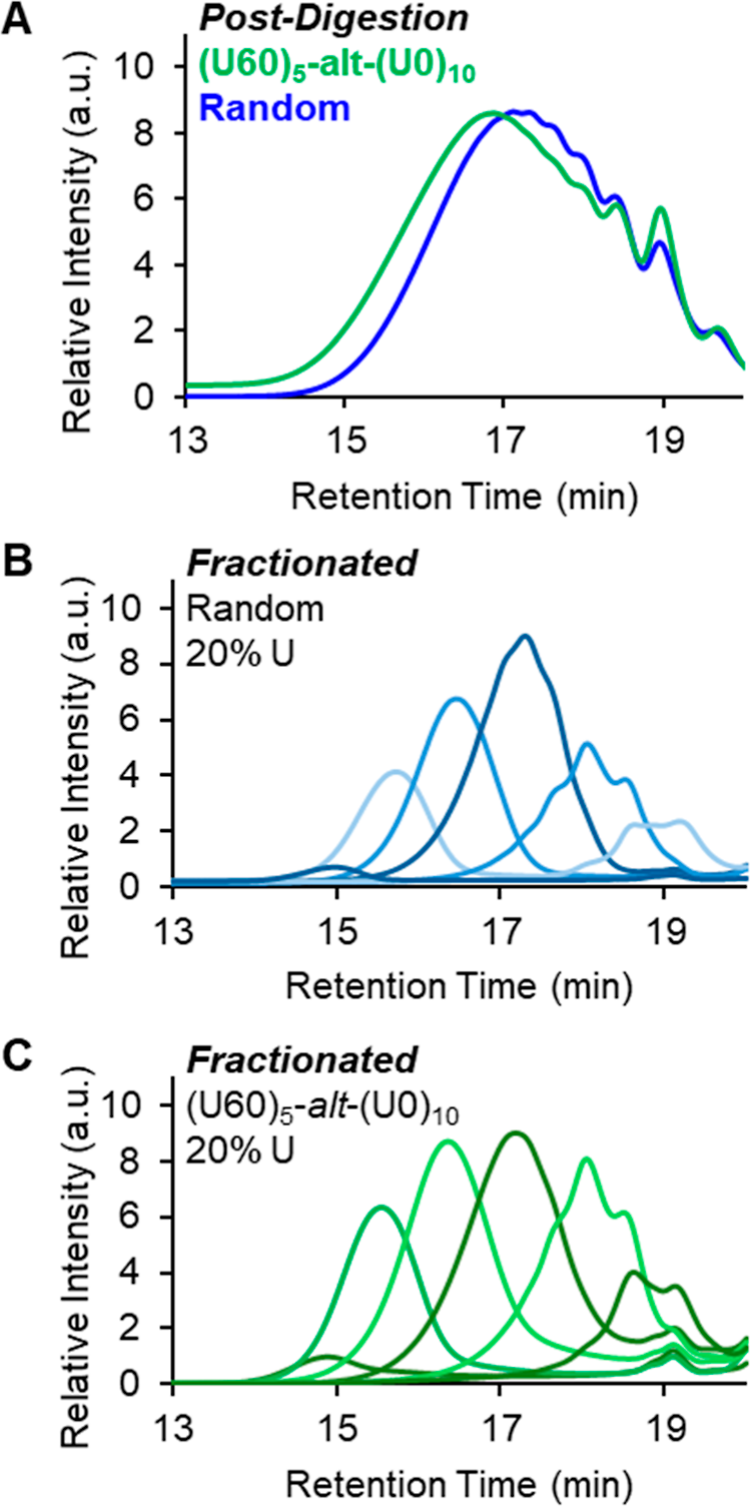
(A) SEC data for postdigestion polymers for Rand­(U20) (blue) and (U60)_5_-*alt*-(U0)_10_ (green). SEC data for postdigestion polymers after fractionation for (B) Rand­(U20) and (C) (U60)_5_-*alt*-(U0)_10_. Postfractionation samples were concentrated to dryness and redissolved in 500 μL THF prior to SEC data collection.

In addition to improving our ability to observe higher molecular weight chains by MALDI, the fractionation also enhances our ability to visualize the differences in fragment profiles between samples with varied sequence distributions (Figure 7A,B,C). In particular, the differences in the SEC data of the postdigestion **Rand­(U20)** and the **(U60)**_**5**_ **-** *alt* **-(U0)**_**10**_ samples are much clearer, which is discussed in detail in the next section.

### Random vs Semi-Sequenced Fragmentation

Based on ^1^H NMR and MALDI analysis, each **U-**containing polymer in this study subjected to metathesis digestion went to greater than 98% completion (Figures 4B and 6). All postdigestion MALDI data were consistent with the supposition that the remaining polymer chains consisted entirely of **GS** repeat units with cleaved **U** end groups. The molecular weight distribution of the postdigestion polymer samples is, therefore, directly related to the **GS** block-length distribution of the predigestion polymer chains.

The SEC-RI data of the fractionated postdigestion polymers **Rand­(U20) trial 2** (Figure 7B) and **(U60)**_**5**_ **-** *alt* **-(U0)**_**10**_ (Figure 7C) have measurably different postdigestion molecular weight distributions despite the two polymers’ comparable predigestion compositions and molecular weights (Table 1). The data suggest that the **(U60)**_**5**_ **-** *alt* **-(U0)**_**10**_ P-S copolymer had a higher relative proportion of longer **GS** block-lengths and shorter **GS** block-lengths compared to its random analog, **Rand­(U20) trial 2**.

### Monte Carlo Simulations

To better understand how monomer feed ratios and oligomer chain lengths influence the sequence distribution in P-S copolymers, Monte Carlo simulations were implemented in Python. Fully commented scripts and the inputs used to generate data are available in the Supporting Information.

### Model Assumptions and Polymerizations

Based on previous experience in our group,^16^ it was assumed that all active end groups of monomers and oligomer chains being modeled had identical reactivities. The experiments also assumed complete oligomerization and no formation of cyclic species. The polymerization step was stopped at 90% completion, a point chosen to ensure modest molecular weight with an average of 10 oligomers per polymer chain, similar to experimental results. All disperse oligomer distributions were approximated using the Flory distribution with stoichiometric imbalance (eq 2)^45^ where where “P_*x*_” is the probability of finding an oligomer containing “*x*” monomers, given the stoichiometric imbalance ratio “*r*”, where “*x*” must be odd

2 $$P_{x,odds}=r^{x/2}\left(1-r\right)r^{-\left(\frac{1}{2}\right)}$$

Once the distribution for an oligomer pool was calculated, the probabilities were scaled up to large integer values that represent the number of chains. The monomer composition and random sequence of each oligomer set were then assigned to be consistent with the targeted monomer feed ratios and an internal alternating AB structure. One oligomer set was an “A” terminated diol (monomer **G**), while the other was a “B” terminated diacid (monomers **U** or **S**).

The P-S copolymerization algorithm is similar to the oligomer generation method. The oligomer sets were treated as macromonomer building blocks to be combined in an alternating fashion. The standard Flory distribution (eq 3), assuming 1:1 functional group stoichiometry, was used to define the polymer chain length distribution, where “*P*_*y*_” is the probability of finding a polymer containing “*y*” oligomers per chain and “*p*” is the extent of reaction.

3 $$P_{y}=\left(1-p\right)p^{\left(y-1\right)}$$

Note: when these oligomers are combined, the random sequences of the individual oligomer chains are preserved in the final copolymer. For clarity, “DP” refers to the number of monomers per chain, where a “monomer” is defined as a single A unit (**G**) or B unit (**U** or **S**), not the full AB combination. In subsequent plots, the AB combination (**GS** or **GU**) will be referred to as a “repeat unit” and explicitly defined as such.

A set of simpler copolymer data sets were also generated, where the disperse oligomer pools were replaced with discrete oligomers containing a defined number of monomers per chain and randomized monomer sequences. The purpose of this data set is to have some examples of simplified postdigestion data, where the effects of confining the digestible monomer to one oligomer pool or the other are more obvious.

Figure 8 shows the chain length distribution of a P-S copolymer (Figure 8B,C) made from alternating diacid (DP̅ 5, 50% **U**) and diol oligomers (DP̅ 10, 0% **U**) (Figure 8A). This copolymer is named **(U50)**_**5**_ **-** *alt* **-(U0)**_**10**_ using the synthesis nomenclature presented previously and has an overall composition such that the diacid monomers are 20% **U** and 80% **S**. It is worth reiterating that all oligomer chains were terminated with stoichiometric imbalance, thus always have an odd number of monomers per chain. When treating the two oligomer pools as macromonomers reacting in an AA/BB step-growth fashion, we expect the number of oligomers per polymer chain to follow the Flory distribution as shown in 8B. In Figure 8C, the relatively large number of odd vs even chains at low DPs are attributed to the presence of monomer and shorter oligomers, particularly from the DP̅ 5 diacid oligomer pool.

**Figure 8:**
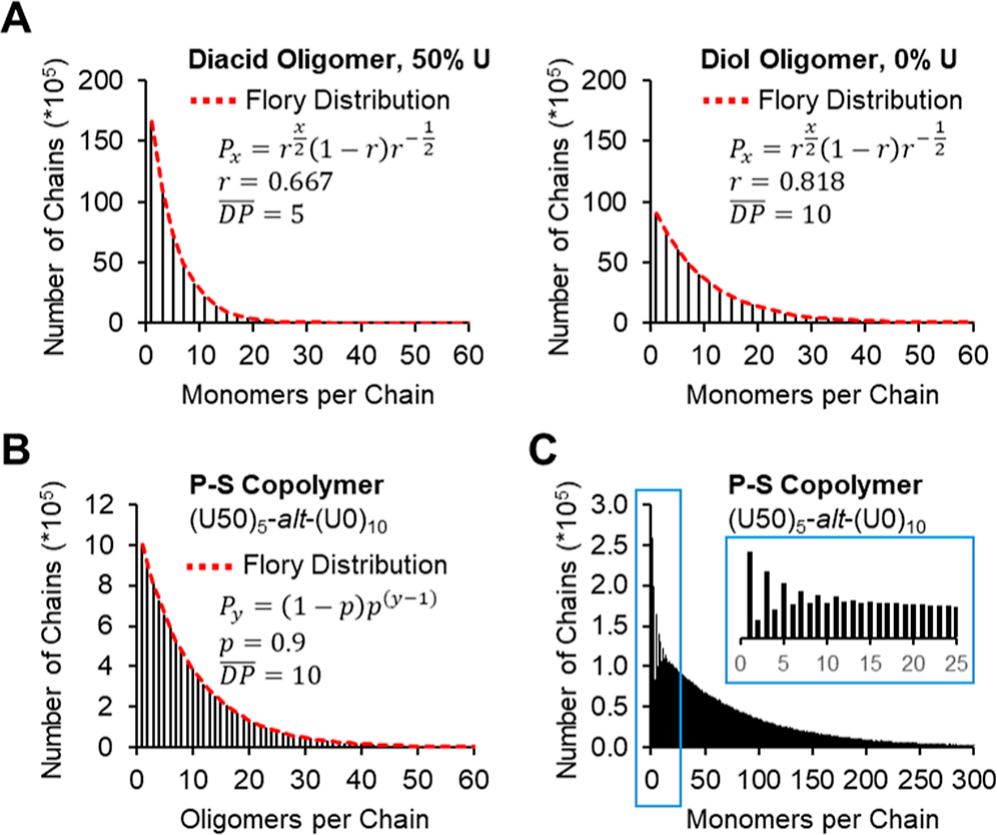
Chain length distributions for the simulated P-S copolymer (U50)_5_-*alt*-(U0)_10_ and its precursor oligomers. The black bars indicate the number of chains of a given length in each data set. The red dashed lines are the theoretical Flory distributions. The diacid and diol oligomers terminated in stoichiometric imbalance (A) were combined in an alternating fashion such that the number of oligomers per chain followed the standard Flory distribution with stoichiometric balance (B). The copolymer chain length distribution is shown in (C).

### In Silico Digestion and Chain Length Distributions

The digestion process was simulated computationally by cleaving the polymer chains at each **U** monomer to produce a population of (**GS**)_*n*_ fragments. The distribution of these fragments is inherently related to the spatial arrangement of the **U** units in the predigestion copolymer. Figure 9 presents a side-by-side comparison of the chain length distributions for **(U50)**_**5**_ **-** *alt* **-(U0)**_**10**_, both before and after the simulated digestion. Figure 9A is a duplicate of Figure 8D, included for reference.

**Figure 9:**
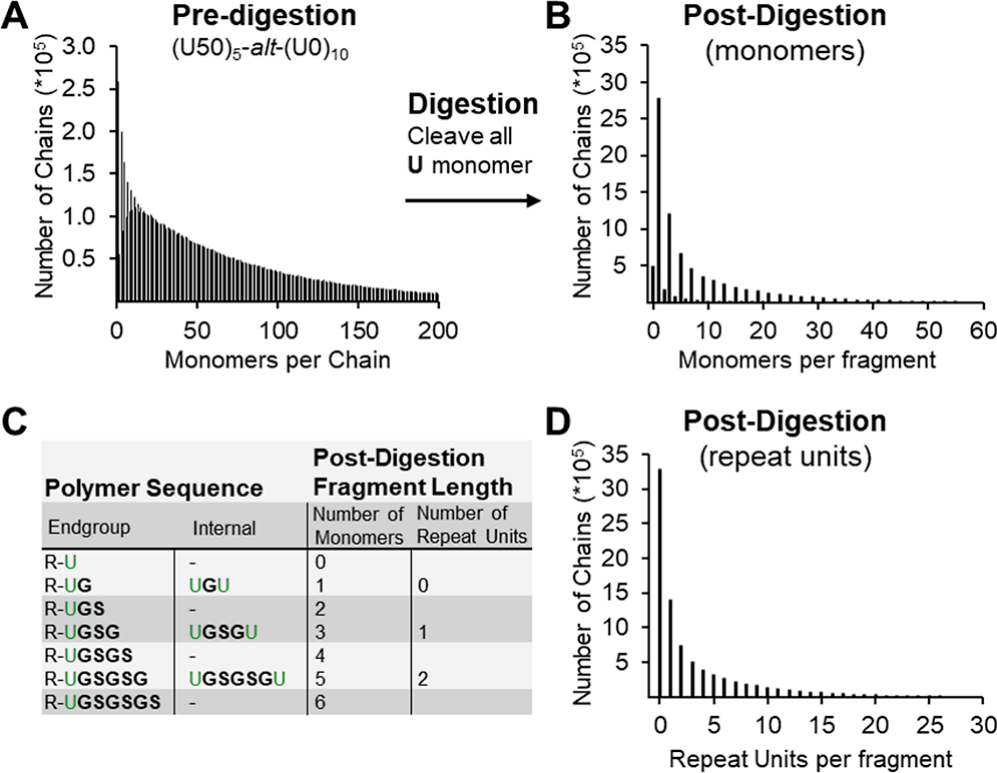
Pre- and postdigestion chain length distributions for the simulated P-S copolymer **(U50)**_**5**_ **-** *alt* **-(U0)**_**10**_ are depicted. (A) The predigestion DP distribution (reproduced from Figure 9D for reference). Chains are then cleaved at **U** monomer sites, and (B) displays the resulting postdigestion DP distribution of fragment lengths, where a low-probability secondary distribution is visible. (C) A table that defines how fragments are binned to convert from monomers to repeat units for the final plot (D). The table also illustrates that fragments containing end groups can be even or odd lengths, while internal fragments must be odd. (D) Postdigestion distribution plotted with respect to the number of repeat units instead of the number of monomers, using the binning rules defined in panel (C).

The postdigestion data, shown in Figure 9B, reveals a chain length distribution where fragments with an odd number of monomers are significantly more common than those with an even number. As illustrated in Figure 9C, the explanation for this pattern is related to the origin of the fragment either from cleavage of two **U** units within the same chain (internal) or a cleavage that generates a fragment which includes an end group. As odd chain lengths can be generated from either internal or end group cleavage, they are more prevalent than even chain lengths which can only arise from internal cleavage.

To simplify the postdigestion data, the chain length was replotted as the number of complete repeat units per chain (Figure 9D), rather than the number of monomers. This was accomplished by combining the abundances of fragments with both even and odd monomer counts that correspond to the same number of complete **GS** repeat units. This method creates a cleaner data plot, as it avoids the secondary distribution caused by end groups and allows for easier comparison of multiple data sets without losing significant information. Any calculations performed refer to the number of monomers per chain, with binning happening only in the final step prior to plotting the data.

A variety of data can be extracted or calculated from the in silico pre- and postdigestion data sets, including the complete DP/MW distributions, the dispersity (Đ), average DP (DP̅), *M*_n_, and *M*_w_. The number-average DP, the length weighted average DP ( ${\overset{®}{DP}}_{l}$ ) and the DP dispersity (Đ_DP_) are analogous to their molecular weight counterparts and are defined in eqs 3–5. When plotting the chain-length distributions in Figure 10, we take two approaches. First, we plot the relative number of chains (N) versus chain length and, second, we plot the length weighted relative number of chains ( $N_{l}$ , eq 6) versus chain length, which places greater significance on chains containing more monomers. As stated earlier, chain lengths are plotted with respect to number of repeat units for visual clarity, avoiding the low intensity secondary distributions that result from end group effects.

4 $$\overset{®}{DP}=\frac{\sum {\left(DP\right)}_{i}\cdot N_{i}}{\sum N_{i}}$$

5 $${\overset{®}{DP}}_{l}=\frac{\sum {\left(DP\right)}_{i}^{2}\cdot N_{i}}{\sum {\left(DP\right)}_{i}N_{i}}$$

6 $${Đ}_{DP}=\frac{{\overset{®}{DP}}_{w}}{{\overset{®}{DP}}_{n}}$$

7 $${\left(N_{l}\right)}_{i}={\left(DP\right)}_{i}\cdot N_{i}$$

**Figure 10:**
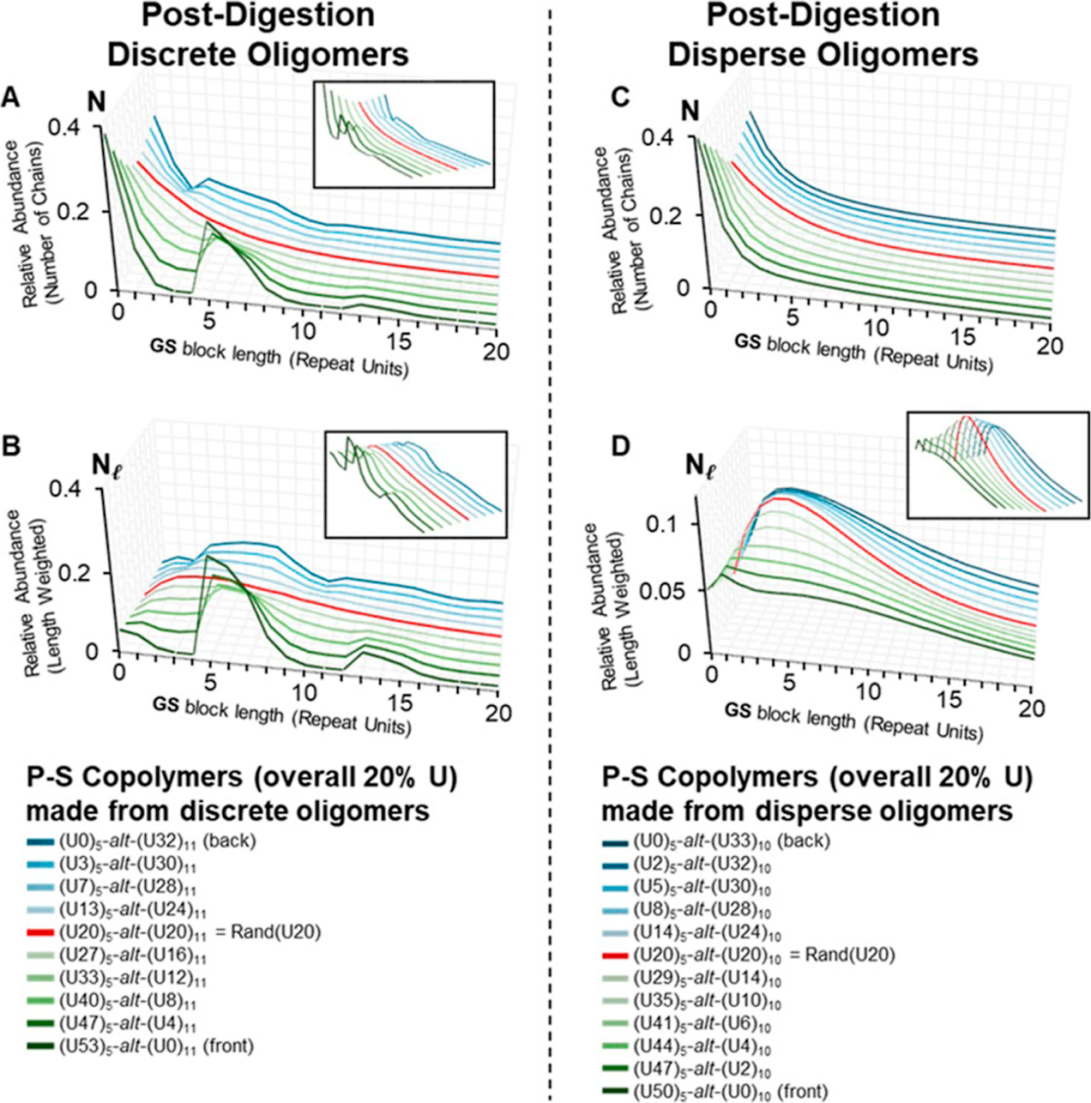
Graphical summary of in-silico postdigestion chain-length distributions of P-S copolymers containing an overall 20% **U** content. (A,B) show results for copolymers made from discrete oligomers (DP 5 and DP 11), while (C,D) show results for copolymers made from disperse oligomers (DP̅s of 5 and 10). For specific compositions (e.g., Panel B, **(U53)**_**5**_ **-** *alt* **-(U0)**_**11**_, dark green, front), certain block-lengths are impossible (e.g., 3–4, 11–12, 19–20 repeat units), while others occur only as low probability end groups. A similar but more subtle pattern, where these absences are replaced with lower probabilities, is evident in the disperse case (D).

### Influence of Parallel-Successive Conditions on Sequence Distribution

The postdigestion block-length distributions are plotted for both the discrete (Figure 10A,B) and disperse (Figure 10C,D) oligomer cases. It should be noted, trends in the data are more obvious in the $N_{l}$ plots. In the discrete oligomer case, the simulations clearly predict that when **U** units are concentrated in one oligomer set, for example **(U53)**_**5**_ **-** *alt* **-(U0)**_**11**_ of Figure 10B, the distribution of **GS** block-lengths becomes broader and bimodal. In this scenario, short blocks, e.g., **(GS)**_**0**_ or **(GS)**_**1**_, and longer blocks, e.g., **(GS)**_**5**_, arise with relatively high probability, while certain intermediate lengths are impossible, e.g., **(GS)**_**3**_ or **(GS)**_**4**_ (See Figure S23 of the Supporting Information for more detail). In the disperse oligomer case, for example **(U50)**_**5**_ **-** *alt* **-(U0)**_**10**_ of Figure 10D, the intermediate lengths are no longer “forbidden” but instead occur at lower probabilities. This bimodality indicates that a typical **GS** repeat unit is more likely to be part of a relatively long block or else an extremely short one, leading to a copolymer with high block-length dispersity.

Conversely, if the **U** units are more evenly shared between the short and long oligomers (e.g., **(U40)**_**5**_ **-** *alt* **-(U10)**_**10**_), the overall dispersity in the block-length distribution is predicted to decrease; intermediate **GS** block-lengths become more probable. When **U** is perfectly balanced between the two oligomer streams, the P-S copolymer assumes a statistically random distribution as seen in **(U20)**_**5**_ **-** *alt* **-(U20)**_**10**_ (disperse oligomers) and **(U20)**_**5**_ **-** *alt* **-(U20)**_**11**_ (discrete oligomers). Note: The copolymers derived from disperse oligomers have subscripts reflecting average DPs whereas the discrete oligomer subscripts must assume a single odd value.

Table 2 summarizes the postdigestion average **GS** block-length, length-weighted average **GS** block-length, and **GS** block-length dispersity values for the in-silico P-S copolymers made from disperse oligomers. The original copolymers each had an average chain length of 75 monomers and 20% **U** content, with 1,000,000 polymer chains per data set. The final data sets only differed in the monomer sequences of individual chains due to the oligomers from which they were made. After the polymers were cleaved at all **U** monomers, the average chain length of the fragments (DP̅ in Table 2) was almost identical across all data sets, with a difference of approximately 0.0001%. This result was expected, as each data set necessarily contained a similar number of chains and total number of monomers after the cleavage process.

**Table 2:** In Silico **GS** Average Block-Lengths^b^

polymer name	**DP̅**^a^	${\overset{®}{DP}}_{l}$ ^a^	Đ_DP_ ^a^
**(U50)**_**5**_ **-** *alt* **-(U0)**_**10**_	3.97	11.94	3.01
**(U47)**_**5**_ **-** *alt* **-(U2)**_**10**_	3.97	11.06	2.79
**(U44)**_**5**_ **-** *alt* **-(U4)**_**10**_	3.97	10.34	2.60
**(U41)**_**5**_ **-** *alt* **-(U6)**_**10**_	3.97	9.77	2.46
**(U35)**_**5**_ **-** *alt* **-(U10)**_**10**_	3.97	8.91	2.24
**(U29)**_**5**_ **-** *alt* **-(U14)**_**10**_	3.97	8.36	2.11
**(U20)**_**5**_ **-** *alt* **-(U20)**_**10**_	3.97	7.99	2.01
**(U14)**_**5**_ **-** *alt* **-(U24)**_**10**_	3.97	8.03	2.02
**(U8)**_**5**_ **-** *alt* **-(U28)**_**10**_	3.97	8.29	2.09
**(U5)**_**5**_ **-** *alt* **-(U30)**_**10**_	3.97	8.54	2.15
**(U2)**_**5**_ **-** *alt* **-(U32)**_**10**_	3.97	8.86	2.23
**(U0)**_**5**_ **-** *alt* **-(U33)**_**10**_	3.97	9.13	2.30

a DP here refers to number of repeat units rather than number of monomers. All table values were calculated directly from postdigestion simulation data according to eqs 3–5.

b However, the values for ${\overset{®}{DP}}_{l}$ and Đ_DP_, which are more sensitive to longer chains, showed significant differences depending on how the **U** monomers were distributed. The data indicate that when **U** monomers were more concentrated in a single oligomer pool, both the postdigestion ${\overset{®}{DP}}_{l}$ and D̵_DP_ increased.

### In-Silico vs Experimental Comparisons

A key result of this study is the qualitative agreement between in silico predictions and experimental digestion data, demonstrating that the postdigestion parallel-successive copolymer **(U60)**_**5**_ **-alt-(U0)**_**10**_ exhibits suppressed midrange chain lengths relative to both high and low molecular weights when compared to its random analog **Rand­(U20)**. As shown in Figure 11, comparisons were made between in-silico predictions and experimentally synthesized and digested polymers. Figure 11B displays the in-silico postdigestion **GS** block-length distributions for **Rand­(U20)** and **(U50)**_**5**_ **-** *alt* **-(U0)**_**10**_, plotted to be more comparable to SEC data. Figure 11A presents the corresponding synthetic postdigestion experimental data from Figure 7, plotted as the deconvolution envelopes of the fractionated samples to emphasize the differences between **(U60)**_**5**_ **-** *alt* **-(U0)**_**10**_ and its random analog. Note: **(U60)**_**5**_ **-** *alt* **-(U0)**_**10**_ is compared with **(U50)**_**5**_ **-** *alt* **-(U0)**_**10**_ because they have the same overall composition of ∼20% U. The name of the experimental polymer is slightly different because it is based on the feed ratio not the final composition as discussed in the synthesis section. The data in both cases are plotted with a normalization of the P–S copolymer to the maximum intensity of the random copolymer.

**Figure 11:**
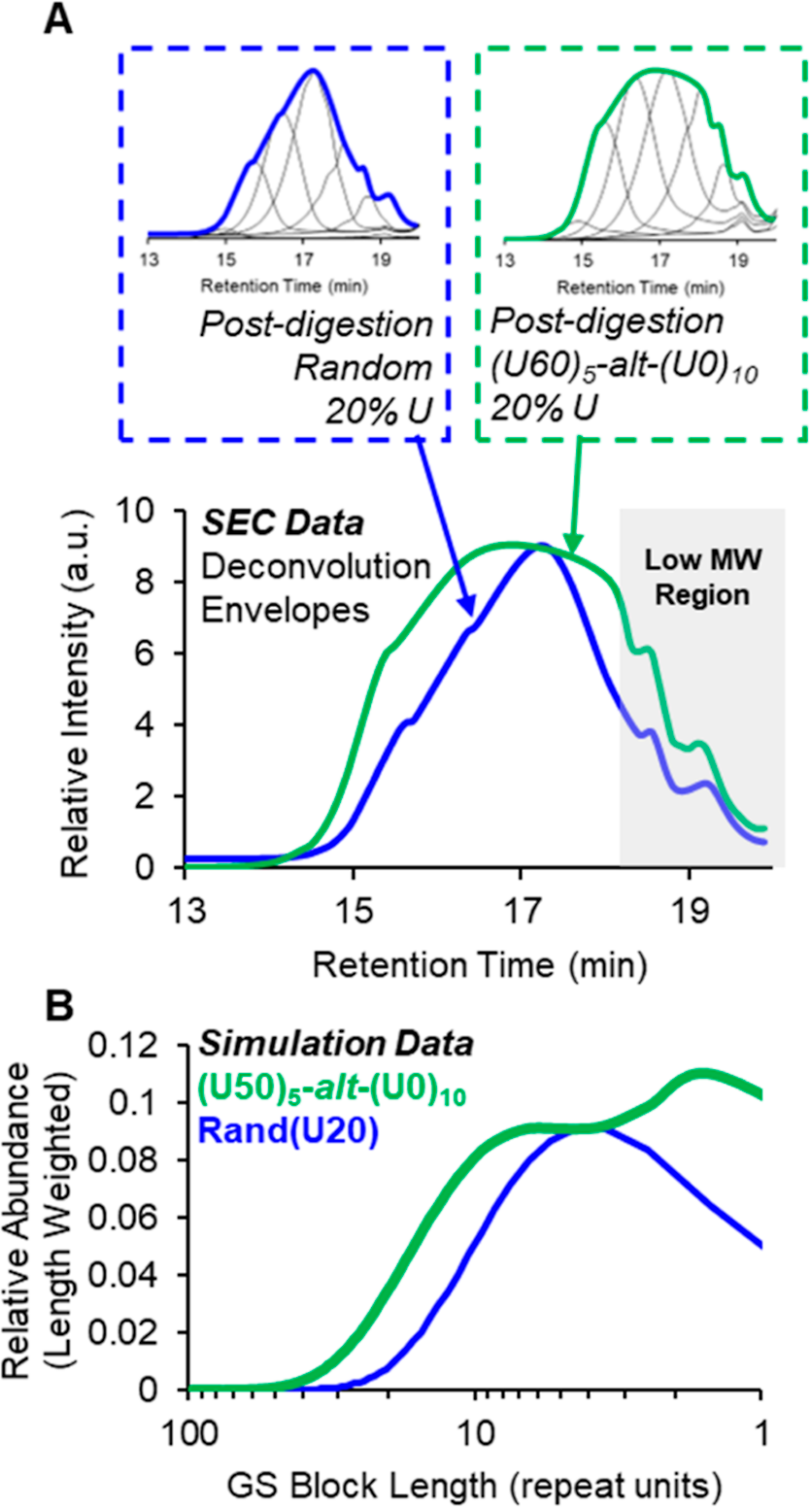
Comparison of postdigestion fractionated polymer SEC data (A) and comparable simulation data (B). All polymers represented in this plot had a predigestion **U** content of 20%. (A) Overlay of the peak deconvolution envelopes for **Rand­(U20)** (blue) and **(U60)**_**5**_ **-** *alt* **-(U0)**_**10**_ (green) from Figure 7 (also inset in A). The deconvolution envelope plot emphasizes the differences between the data sets. (B) Overlay of the in-silico postdigestion molecular weight data for **Rand­(U20)** (blue) and **(U50)**_**5**_ **-** *alt* **-(U0)**_**10**_ (green). The *x*-axis is plotted on the logarithmic scale and inverted such that lower DP values are on the right, which is consistent with the presentation of SEC data. The low MW region of the experimental SEC data in (A) has a lower intensity than would be expected due to predigestion precipitation (gray box).

The predicted pattern, an increase in both high- and low-molecular-weight species with a corresponding depletion of midrange fragments in the P-S copolymer, is clearly evident in the experimental SEC data. Such a cleavage profile could only arise if the digestible **U** monomers were preferentially located in the shorter oligomer chains, thereby reducing the likelihood of certain sequence arrangements and making a truly random **U** distribution impossible. Importantly, the predigestion SEC traces for both samples exhibited the expected shape and dispersity characteristics of step-growth polymers, confirming that these postdigestion differences originate from microstructural sequence variations, not from differences in polymerization efficiency or initial MW distribution.

While the trends are the same for the simulated and experimental data sets, some important experimental considerations prevent direct quantitative comparisons. The most important difference can be attributed to the precipitation of the synthetic polymers. Experimentally, the copolymers were precipitated after synthesis to remove impurities that could interfere with metathesis digestion. Precipitation leads to an inevitable loss of low molecular weight polymer chains prior to digestion, which is ultimately manifested in the postdigestion data as well. No attempt was made to represent the effects of precipitation in the simulations, however, as we felt that including the low molecular weight data was helpful and any exclusion would be somewhat arbitrary. As a result, the experimental SEC data are necessarily shifted to higher MWs and do not include the lower MW chains. This issue is identified in Figure 11A by the gray box which indicates the region in which we expect the intensities to be depressed due to precipitation.

Another factor that is related to the interpretation of the experimental data is our use of polymer fractionation. Although it can clearly be seen in the original that the midrange molecular weights are suppressed relative to the higher MWs, the differences in the postdigestion SEC data were not as apparent without the fractionation step which makes it much easier to visualize. Our choice of 6 fractions, is sufficient to differentiate the midrange from the low and high. As we were careful to use identical fractionation conditions for both samples, we believe that the comparison is valid. We do acknowledge, however, that the need for this additional step represents a limitation of our methodology.

## Discussion

### Implications for Microstructure and Properties

In this paper we showed, using Monte Carlo simulations, that a parallel-succussive methodology can be used to create a variety of semirandom microstructures, distinct from purely random copolymers with the same overall composition. We were also able to demonstrate that such systems could be prepared experimentally. These P-S copolymers, which included a bespoke cleavable unit, were then successfully digested to give a set of fragments whose analysis provided a clearer view of the microstructure than could be obtained using conventional NMR and mass spectrometry techniques.

The experimental digestion profiles closely match the trends predicted by our simulations. We have clear evidence that the **Rand­(U20)** and **(U60)**_**5**_ **-** *alt* **-(U0)**_**10**_ copolymers were successfully synthesized and fully digested. The resulting postdigestion molecular weight distributions that were measured, therefore, reflect the distribution of the **U** monomer in each polymer. As predicted, we can see the postdigestion **(U60)**_**5**_ **-** *alt* **-(U0)**_**10**_ polymer has an increased dispersity compared to its random analog (1.8 vs 1.6, Table 1). Postdigestion fractionation of each polymer shows that both high and low molecular weight chains are present in higher proportions in the **(U60)**_**5**_ **-** *alt* **-(U0)**_**10**_ sample compared to **Rand­(U20)** as shown in Figures 7 and 11A.

Our Monte Carlo simulations offer valuable insight into how parallel-successive copolymerization conditions influence monomer sequence microstructure in semirandom copolymers. The transition from blockier, bimodal block-length monomer distributions to statistically distributed monomers is obvious when comparing extreme cases but becomes subtle with minor adjustments as shown in Figure 10. These observations will help guide future design of copolymers. It is also worth noting that these calculations are generally applicable to other step-growth polymers, potentially expanding the future application space.

Importantly, the olefin in the **U** monomer that was used to facilitate sequence characterization could easily be substituted with a variety of property enhancing functional groups. Drug delivery, for example, could be facilitated by tuning the drug molecule distribution throughout a polymer backbone. Similarly, the distribution of property altering functional groups could be used to control material properties, as has been already demonstrated in polyurethanes.

## Conclusions and Outlook

From the combined experimental and in-silico approaches described here, we conclude that parallel-successive (P-S) copolymerization is a useful approach to synthesizing semirandom polymers with tunable functional group distributions. We utilized a model system containing a cleavable monomer unit to allow us to infer the sequence distribution of P-S copolymers, confirming the general trends observed in the computational data. This general approach can be used to predict the monomer feed ratios required to achieve specific sequence patterns in comparable P-S copolymers where direct postsynthetic sequencing is limited or impossible and could be used to create a range of tunable semisequenced copolymers for applications. We anticipate that the P-S approach will be particularly valuable for designing bioengineering-relevant polymers with clustered functionality as it has been shown previously that material interactions and performance can depend on how functional groups are distributed along the chain.^46–48^ Such polymers could be created using the chemistry described herein if the olefin functionality was replaced by a functional group relevant to a particular application.

## Supplementary Material



## Notes

Synthetic experimental and characterization details, digestion protocols, and a description of the modeling methodology (PDF)
