# Supplementary material for: Characterization of Sequence Distributions in Random and Semi-Random Copolymers
Source: Macromolecules. 2026 Mar 3;59(6):3722–33. doi: 10.1021/acs.macromol.5c01799 (PMC13038135; doi:10.1021/acs.macromol.5c01799)
Supplement: Supplementary file 1 [file ma5c01799_si_001.pdf]

Characterization of Sequence Distributions in Random and Semi-Random Copolymers

Supporting Information

TABLE OF CONTENTS

EXPERIMENTALS **S1-28**

MATERIALS AND INSTRUMENTATION S1

MONOMERS S3-18

G (GAG) *S3*

S (LSL) *S11*

U (LUL) *S15*

RANDOM COPOLYMERS S19

PARALLEL-SUCCESSIVE COPOLYMERS S23

POLYMER DIGESTION S26

MONTE CARLO SIMULATIONS **S29-42**

POLYMER GENERATION ALGORITHM OVERVIEW S29

POLYMER GENERATION INPUTS AND SOURCE CODE S31

DATA EXTRACTION OVERVIEW S38

DATA EXTRACTION SOURCE CODE S39

GS BLOCK-LENGTHS IN DISCRETE OLIGOMER CASE S42

Experimentals

Materials. All experiments were carried out in oven-dried or flame-dried glassware under an atmosphere of N_2 using standard Schlenk line techniques. 4-(dimethylamino)pyridinium 4-toluenesulfonate (DPTS)[1] and benzyl-(S)-lactate (Bn-L)[2,3] were prepared according previously-published protocols. Dichloromethane (DCM, Fisher) and ethyl acetate (EtOAc, Fisher) were purified by a Solvent Dispensing System by J. C. Meyer by passing over two columns of neutral alumina. 1,2-dichloroethane (DCE) was purchased from Fisher Scientific and dried over CaH_2 and distilled before using. All other chemicals were used without further purification. N,N'-dicyclohexylcarbodiimide (DCC), N,N'-diisopropylcarbodiimide (DIC), 2,2-dimethy-1,3-propanediol and tetrabutylammonium fluoride (TBAF) were purchased from Acros Organics. Succinic acid, triethylamine (TEA) and styrene were purchased from Sigma-Aldrich. Trans-3-hexanedioic acid and methyl glycolide (MeG) were purchased from TCI. Grubbs 2nd generation catalyst (G2) was purchased from AK Scientific. 10% by weight palladium on carbon (Pd/C), palladium (II) acetate ($Pd(OAc)_2$) and triethylsilane were purchased from Aldrich. 4-dimethylaminopyridine (DMAP) and tert-butyldiphenylsilyl chloride (TBDPSCl) were purchased from Oakwood. Deuterated NMR solvents ($CDCl_3$ and DMSO-d_6) were purchased from Cambridge Isotope Laboratories. Methanol (MeOH) and diethyl ether were purchased from Fisher Scientific. Anhydrous, inhibitor-free Tetrahydrofuran (THF, $\geq$99.9%) was purchased from Alfa Aesar. Column chromatography was performed using Sorbent Technologies 60 Å, 40-63 µm standard grade silica.

NMR Spectroscopy. ^{1}H (400 and 500 MHz) and ^{13}C (100 and 125 MHz) NMR spectra were obtained using Bruker spectrometers and are reported as δ values in ppm relative to the reported solvent. Splitting patterns are abbreviated as follows: singlet (s), doublet (d), triplet (t), quartet (q), multiplet (m), broad (br), and combinations thereof.

Size Exclusion Chromatography. Molecular weights and dispersities were obtained on a TOSOH HLC-8320GPC EcoSEC equipped with two columns (TSKgel-G3000H, TSKgel-G3000H). A mobile phase of THF inhibited with 0.025% butylated hydroxytoluene at 50 °C with a flow rate of 1 mL/min was used, reported molecular weights were obtained with a refractive index detector (TOSOH) and are relative to polystyrene standards (90, 50, 30, 9, 5, 2.5 kDa).

MALDI-ToF MS. MS spectra were obtained on a Bruker ultrafleXtreme MALDI-ToF instrument. An accelerating voltage of 20 kV was applied, and spectra were obtained in reflection positive mode (500 shots). The polymers were dissolved in THF to yield a concentration of 1 mg/mL. Fractionated polymer solutions were used as collected to make those samples. Sodium trifluoroacetate (NaTFA) was used as the catonization agent and was dissolved in THF to form a 1 mg/mL solution. The matrix was DCTB in THF as a 40 mg/mL solution. The three solutions were combined in a volume ratio of 2:3:2 (polymer: DCTB: NaTFA). The solution was then drop cast onto a 100-well MALDI plate and allowed to dry before analysis. Spectra were analyzed using Bruker flexAnalysis software package.

Polymer Fractionation. An SEC equipped with an automated fractionator (Varian ProStar) was used to perform analytical scale fractionation of post-digestion polymer samples to facilitate characterization. All samples were auto-fractionated using the same time-based method. The fraction size in all cases was 1 minute (1 mL/min). The fractionation was repeated five times for each sample into the same set of collection tubes. The fractions were then concentrated to dryness and redissolved in 500 uL of THF before being reinjected into the SEC. This was done to ensure that the relative amount of material collected in each fraction was reflected in the relative concentration (mg/mL) of each sample.

Monte Carlo Simulations. All simulations were performed using Python version 3.9.13 on the University of Pittsburgh Center for Research Computing and Data's H2P computing cluster.

Synthesis of GAG ("G" in main text)

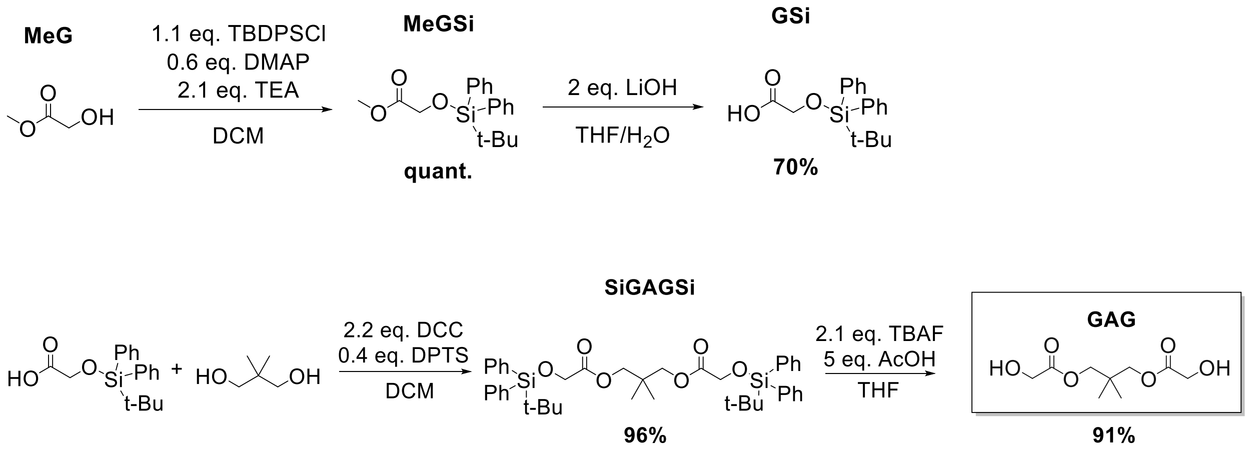

MeGSi. MeG (15.9 g, 153 mmol) and triethylamine (32.5 g, 321 mmol) were added via syringe to a stirring solution of dry DCM (500 mL). DMAP (10.7 g, 87.9 mmol) was added via funnel under heavy N_2 flow. The reaction was chilled over an ice bath before adding TBDPSCl (47.5 g, 173 mmol) dropwise over 25 minutes. Once the addition was complete, the reaction was allowed to return to room temperature and the reaction was stirred for 16 h. The mixture was then filtered, and the organic layer was washed with 1 M HCl (3 x 200 mL) and brine (1 x 200 mL). The organic layer was dried over $MgSO_4$, filtered and concentrated to produce the desired product in quantitative yield with some silyl impurity contamination (50.3 g). ^{1}H NMR (400 MHz, $CDCl_3$) δ 7.28 (m, 4 H), 7.35 (m, 6H), 4.25 (s, 2 H), 3.68 (s, 3H), 1.09 (s, 9 H); ^{13}C NMR (100 MHz, $CDCl_3$) δ 171.8, 135.7, 132.9, 130.0, 127.9, 62.6, 51.81, 26.8, 19.4; HRMS (ASAP) calcd. mass 329.1573, found 329.1556.

MeGSi

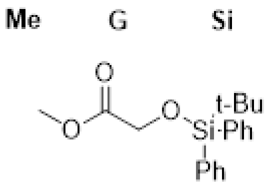

**^{1}H-NMR** (400 MHz, $CDCl_3$)			
δ (ppm)	Mult. (J (Hz))	Int.	Assignment
1.09	s	9	t-Bu (Si)
3.68	s	3	**CH₃** (Me)
4.25	s	2	**CH₂** (G)
7.37-7.45	m	6	Aromatic (Si)
7.67-7.70	m	4	Aromatic (Si)

**^{13}C-NMR** (100 MHz, $CDCl_3$)	
δ (ppm)	Assignment
19.40	**C**(CH_3)$_3$ (Si)
26.80	C(**CH₃**)$_3$ (Si)
51.81	**CH₃** (Me)
62.26	**CH₂** (G)
127.92	Aromatic
130.04	Aromatic
132.91	Aromatic
135.72	Aromatic
171.84	Carbonyl

**HRMS** (APCI)
Composition
$C_{19}H_{24}O_3Si$
Calc.
$[M + H]^+$
329.1573
Found
$[M + H]^+$
329.1556
Delta (ppm)
-5.2

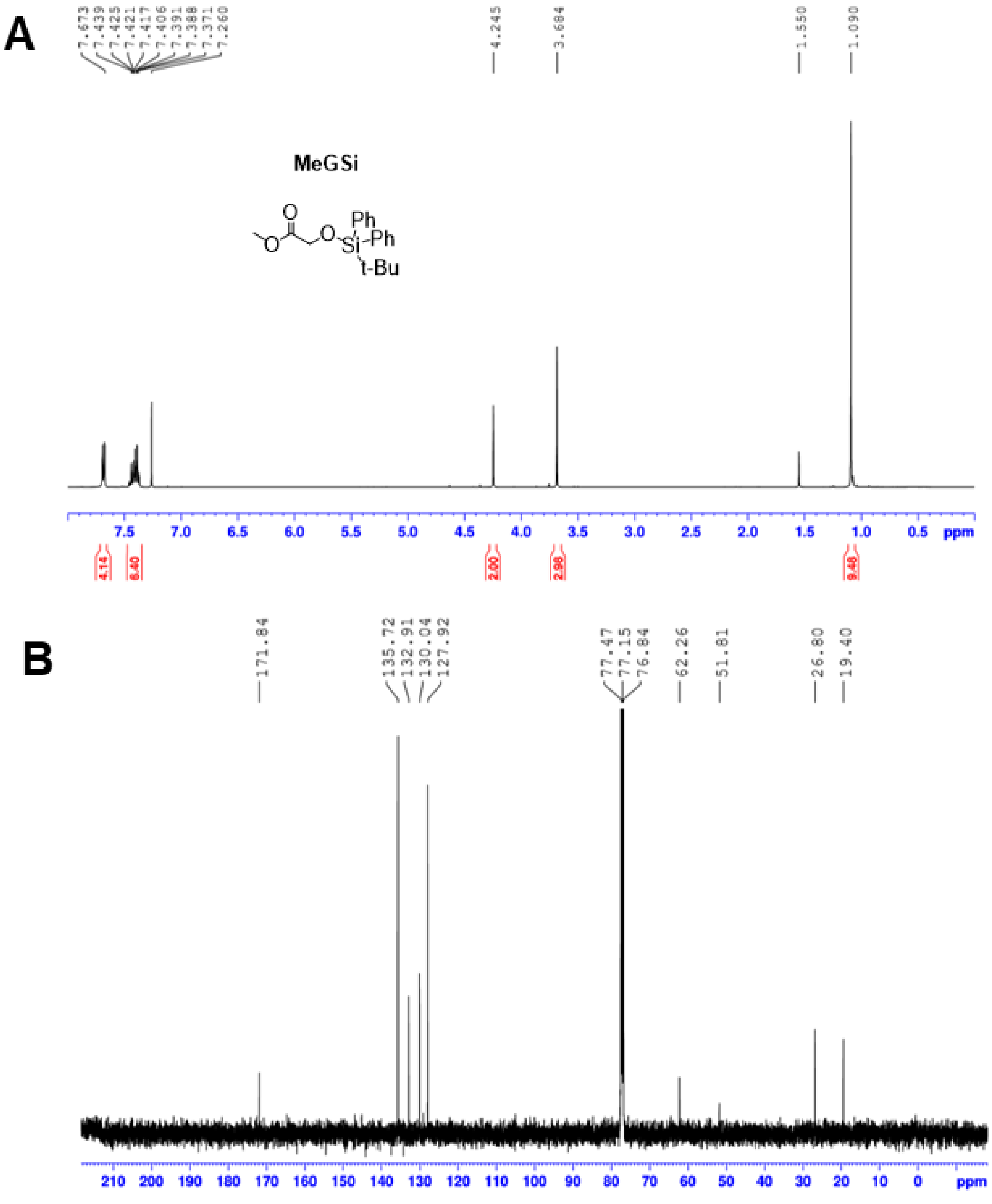

Figure S1. NMR spectra of MeGSi. A) ^{1}H NMR, 400 MHz, $CDCl_3$ B) ^{13}C NMR, 100 MHz, $CDCl_3$

GSi. MeGSi (35.5 g, 108 mmol) was dissolved in THF (800 mL). $LiOH \cdot H_2O$ (9.0 g, 210 mmol) was dissolved in deionized water (800 mL). The MeGSi solution was chilled on an ice water bath and the LiOH solution was added dropwise over 1 hour while stirring. The THF was removed under reduced pressure, and 400 mL brine was added to the remaining aqueous solution. This was extracted with diethyl ether (3 x 400 mL) to remove unreacted starting material. Another LiOH solution was prepared by dissolving $LiOH \cdot H_2O$ (9.0 g, 210 mmol) in deionized water (800 mL). The combined ether layers were extracted with the 800 mL LiOH solution and discarded. The aqueous layers were combined and acidified with approximately 600 mL of 1 M HCl over a 10-minute period. The acidified aqueous solution was extracted with diethyl ether (3 x 400 mL) to isolate the product. The latter set of ether extractions were combined, dried over $MgSO_4$, filtered and concentrated under reduced pressure to yield the product as a pure oil (23.7 g, 70%). ^{1}H NMR (300 MHz, $CDCl_3$) δ 7.65 (m, 4 H), 7.44 (m, 6 H), 4.23 (s, 2 H), 1.11 (s, 9 H)

GSi

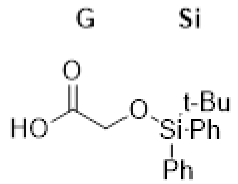

**^{1}H-NMR** (300 MHz, $CDCl_3$)			
δ (ppm)	Mult. (J (Hz))	Int.	Assignment
1.11	s	9	t-Bu (Si)
4.23	s	2	C**H₂** (G)
7.38-7.48	m	6	Aromatic (Si)
7.63-7.67	m	4	Aromatic (Si)

**^{13}C-NMR** (100 MHz, $CDCl_3$)	
δ (ppm)	Assignment
19.13	**C**$(CH_3)_3$ (Si)
26.70	C(**C**$H_3)_3$ (Si)
62.03	**C**H_2 (G)
128.16	Aromatic
130.47	Aromatic
131.40	Aromatic
135.38	Aromatic
171.21	Carbonyl

**HRMS** (ESI)
Composition
$C_{19}H_{24}O_3Si$
Calc.
$[M + H]^+$
Found
$[M + H]^+$
Delta (ppm)

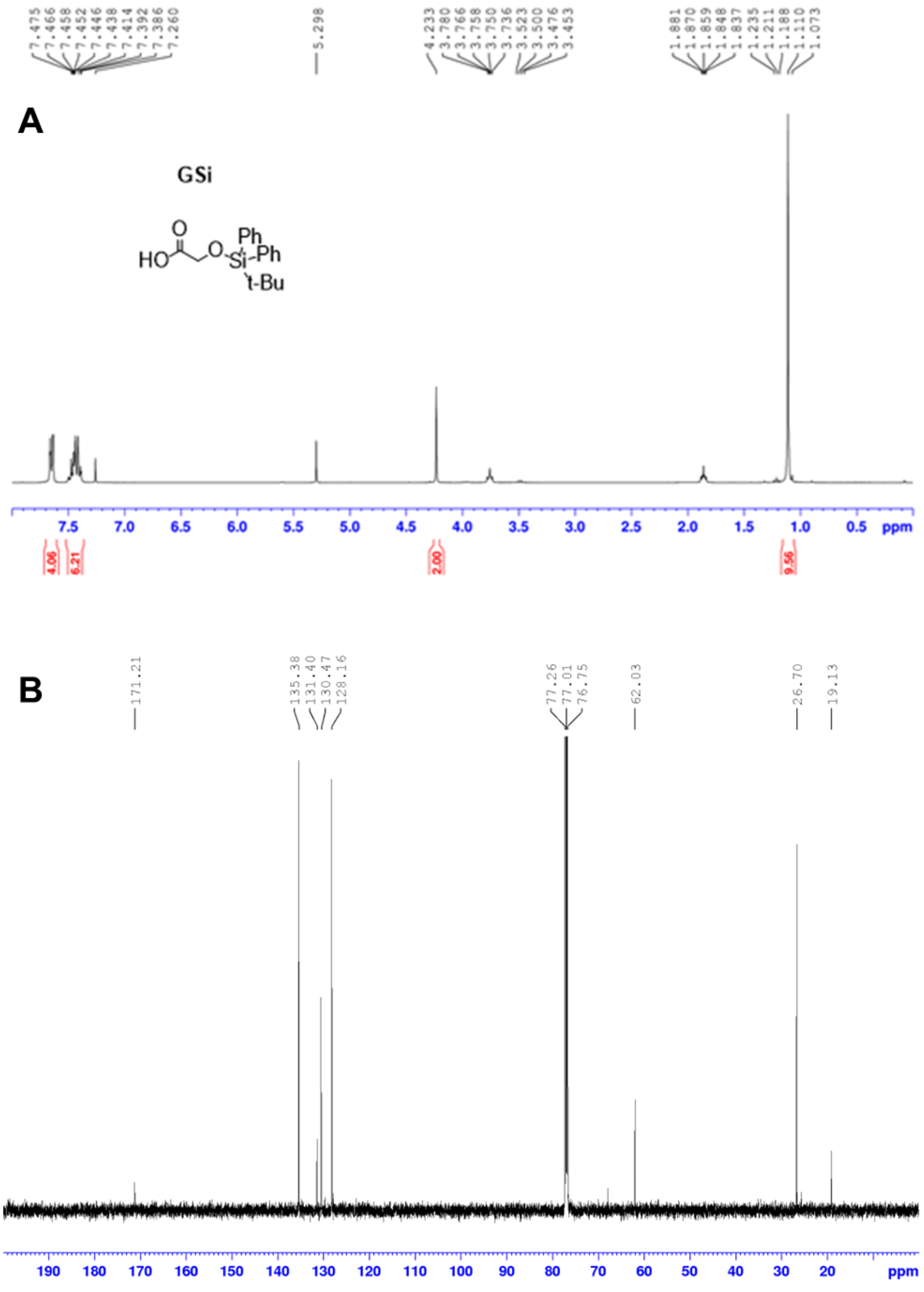

Figure S2. ^{1}H NMR spectrum of GSi. A) ^{1}H NMR, 300 MHz, $CDCl_3$ B) ^{13}C NMR, 100 MHz, $CDCl_3$

SiGAGSi. GSi (1.46 g, 4.6 mmol) and 2,2-dimethyl-1,3-propanediol (0.23 g, 2.2 mmol) were added to a stirring solution of dry DCM (110 mL). DPTS was added (0.26 g, 0.9 mmol). DCC (1.1 g, 5.4 mmol) was added and the reaction was allowed to stir for 18 h. The reaction was then filtered and concentrated under reduced pressure. The crude material was purified by flash chromatography (SiO_2, 10-15% ethyl acetate in hexanes) to produce the pure oil (1.47 g, 96%). ^{1}H NMR (400 MHz, $CDCl_3$) δ 7.68 (m, 8 H), 7.40 (m, 12 H), 4.24 (s, 4 H), 3.87 (s, 4 H), 1.08 (s, 18 H), 0.87 (s, 6 H); ^{13}C NMR (125 MHz, $CDCl_3$) δ 171.2, 135.7, 132.9, 130.1, 128.0, 69.3, 62.3, 34.9, 26.8, 21.7, 19.4

SiGAGSi

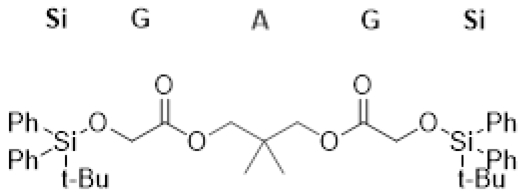

^{1}H-NMR (500 MHz, $CDCl_3$)

δ (ppm)	Mult. (J (Hz))	Int.	Assignment
0.87	s	18	t-Bu (Si)
1.08	s	6	**CH_3** (A)
3.87	s	4	**CH_2** (A)
4.24	s	4	**CH_2** (G)
7.35-7.45	m	12	Aromatic (Si)
7.66-7.69	m	8	Aromatic (Si)

^{13}C-NMR (125 MHz, $CDCl_3$)

δ (ppm)	Assignment
19.38	**C**$(CH_3)_3$ (Si)
21.69	C(**CH_3**)$_3$ (Si)
26.80	C(**CH_3**)$_2$ (A)
34.90	**C**$(CH_3)_2$ (A)
62.27	**C**H_2 (G)
69.32	**C**H_2 (A)
127.96	Aromatic
130.07	Aromatic
132.89	Aromatic
135.70	Aromatic
171.20	Carbonyl

HRMS (ESI)

HRMS (ESI)
Composition
$C_{41}H_{52}O_6Si_2$
Calc.
$[M + H]^+$
697.33752
Found
$[M + H]^+$
697.33778
Delta (ppm)
0.38

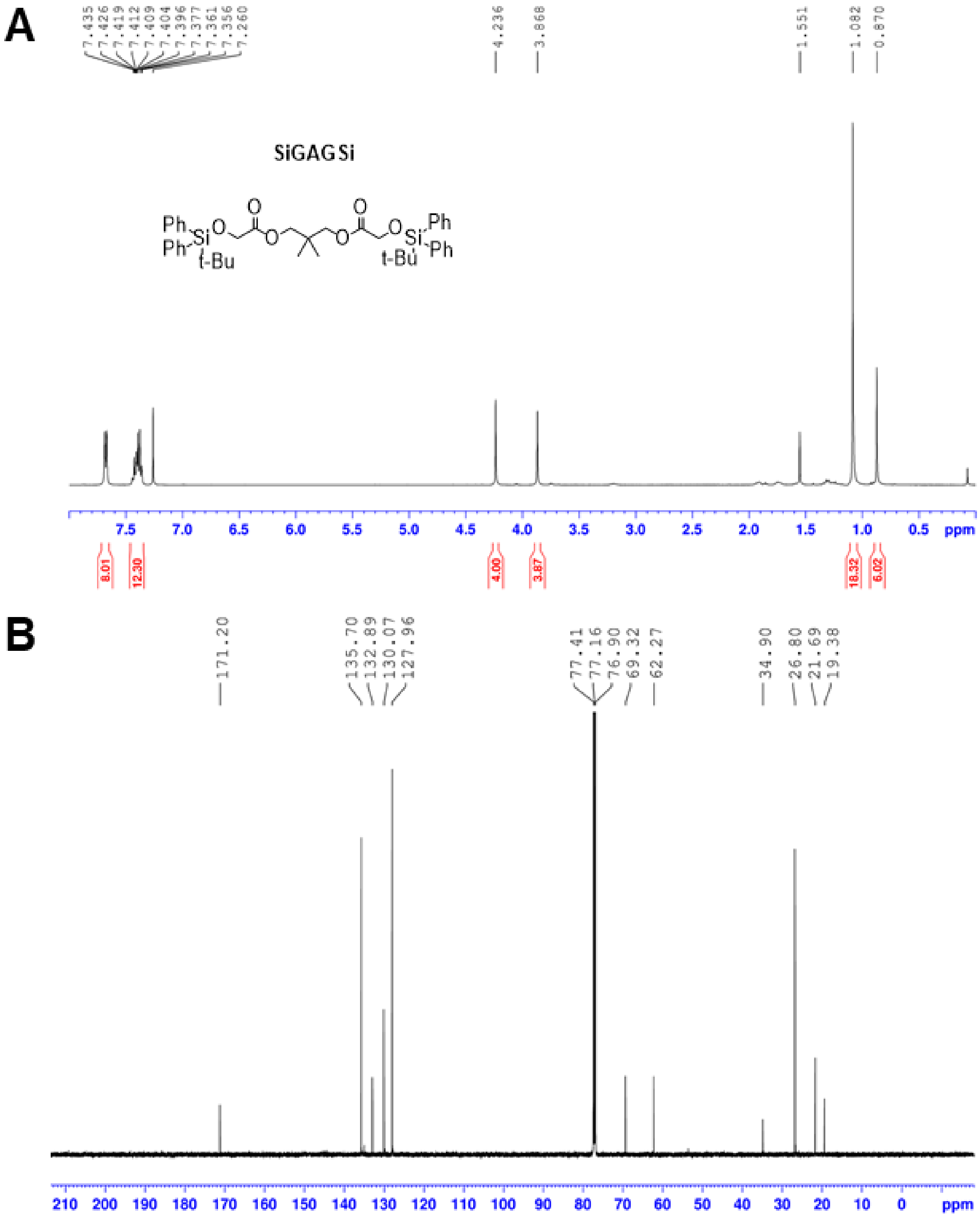

Figure S3. NMR spectra of SiGAGSi. A) ^{1}H NMR, 500 MHz, $CDCl_3$ B) ^{13}C NMR, 125 MHz, $CDCl_3$

GAG. SiGAGSi (2.8 g, 4.06 mmol) was dissolved in 38 mL dry THF. Acetic acid (1.2 mL, 20 mmol) was added while stirring, followed by 1 M TBAF in THF (8.1 mL, 8.1 mmol). The reaction was stirred for 2 h, then placed in a freezer for 3 hours to precipitate most of the TBAF out of the reaction mixture. The TBAF was filtered off and the reaction was concentrated under reduced pressure. The crude oil was purified by flash chromatography (SiO_2, 25% EtOAc in hexanes to remove silyl impurities and mono-deprotected material, 50% EtOAc in hexanes to isolate product) to yield the purified white solid (0.82 g, 91%). ^{1}H NMR (400 MHz, $CDCl_3$) δ 4.18 (s, 4 H), 4.02 (s, 4 H), 2.38 (br, 2 H), 0.99 (s, 6 H); ^{13}C NMR (125 MHz, $CDCl_3$) δ 173.4, 70.0, 60.6, 35.0, 21.8; HRMS (ASAP) calcd. mass 221.1025, found 221.1014

GAG (“G” in main text)

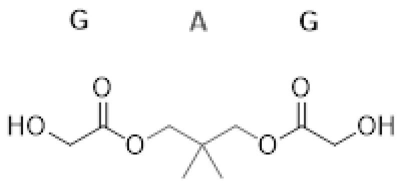

^{1}**H-NMR** (500 MHz, $CDCl_3$)			
δ (ppm)	Mult. (J (Hz))	Int.	Assignment
0.99	s	18	**CH_3** (A)
2.38	br	2	**OH** (A)
4.02	s	4	**CH_2** (A)
4.18	s	4	**CH_2** (G)

^{13}**C-NMR** (125 MHz, $CDCl_3$)	
δ (ppm)	Assignment
21.75	C(**C**H$_3$)$_2$ (A)
35.04	**C**(CH$_3$)$_2$ (A)
60.63	**C**H$_2$ (G)
69.95	**C**H$_2$ (A)
173.39	Carbonyl

**HRMS** (APCI)
Composition
$C_9H_{16}O_6$
Calc.
$[M + H]^+$
221.1025
Found
$[M + H]^+$
221.1014
Delta (ppm)
-5.0

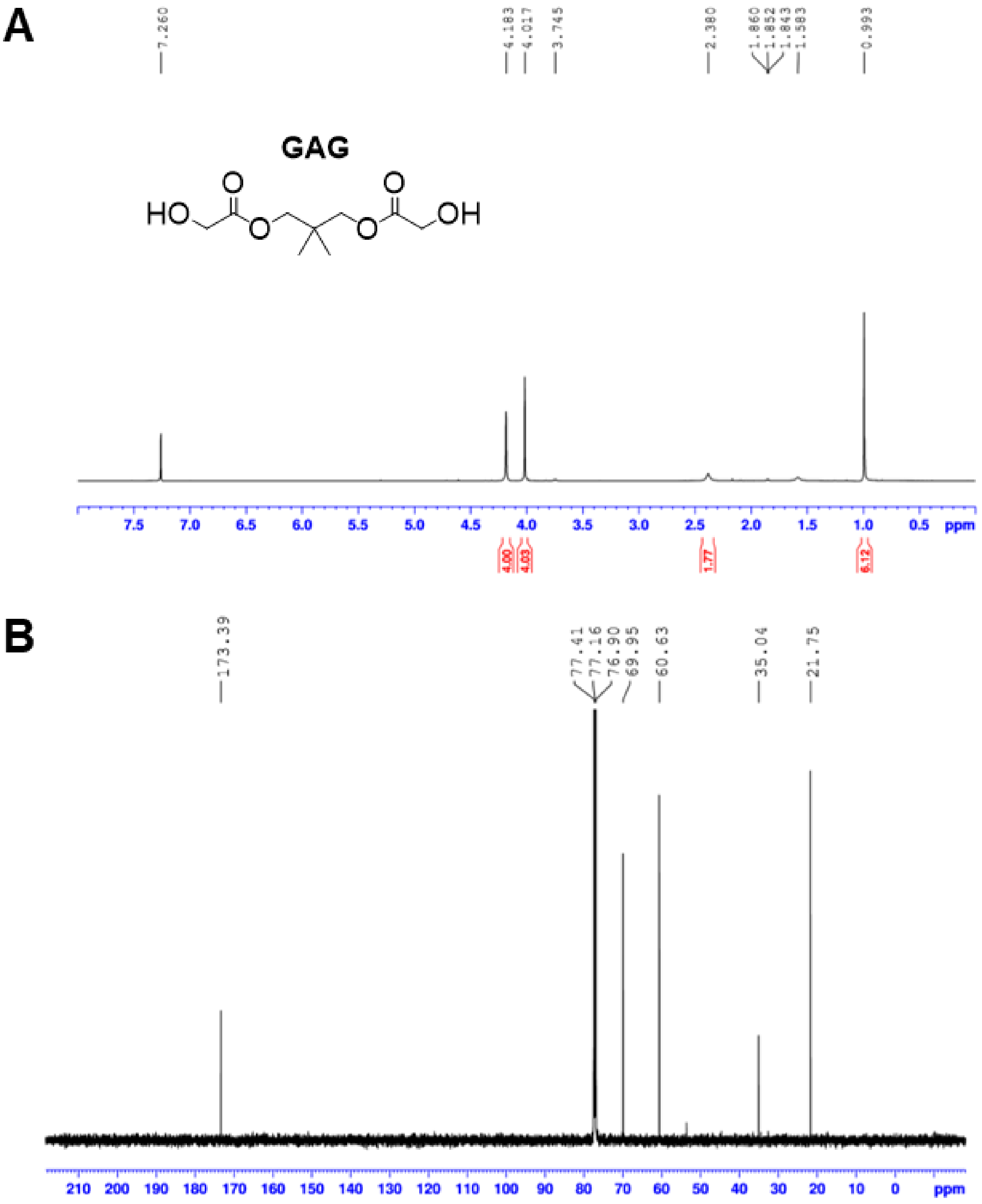

Figure S4. NMR spectra of **GAG** ("G" in main text). A) ^{1}H NMR, 500 MHz, $CDCl_3$ B) ^{13}C NMR, 125 MHz, $CDCl_3$

Synthesis of LSL ("S" in main text)

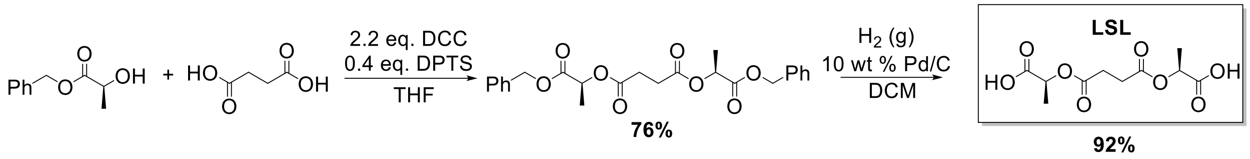

BnLSLBn. BnL (2.1 g, 11.8 mmol) and succinic acid (0.60 g, 5.0 mmol) were added to a stirring solution of THF (210 mL). The reaction was placed on a 50 °C oil bath and once the succinic acid was completely dissolved, DPTS was added (0.34 g, 1.1 mmol). The DPTS catalyst did not completely dissolve. DCC (2.7 g, 12.9 mmol) was added and the reaction was allowed to stir for 24 h at 50 °C. Additional succinic acid (0.77 g, 6.5 mmol) and DCC (1.9 g, 8.7 mmol) were added and the reaction was stirred for an additional 8 h at 50 °C to consume any remaining BnL. The reaction was then filtered and concentrated under reduced pressure. The crude material was purified by flash chromatography (SiO_2, 10-15% EtOAc in hexanes) to produce the pure oil (2.0 g, 76 %). ^{1}H NMR (400 MHz, $CDCl_3$) δ 7.35 (m, 10 H), 5.16 (m, 6 H), 2.73 (m, 4 H), 1.49 (d, J = 7.0 Hz, 3 H); ^{13}C NMR (125 MHz, $CDCl_3$) δ 171.6, 170.7, 135.5, 128.8, 128.6, 128.3, 69.0, 67.2, 28.9, 17.0; HRMS (ESI) calcd. mass 443.17004, found 443.17224.

BnLSLBn

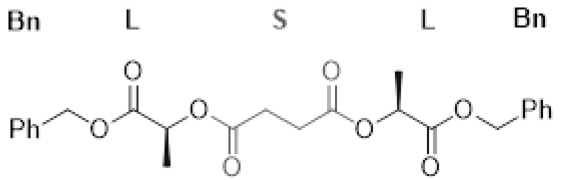

**^{13}C-NMR** (100 MHz, $CDCl_3$)	
δ (ppm)	Assignment
17.00	**C**H_3 (L)
28.87	**C**H_2 (S)
67.15	**C**H_2 (Bn)
69.03	**C**H (L)
128.27	Aromatic
128.56	Aromatic
128.76	Aromatic
135.47	Aromatic
170.65	Carbonyl
171.67	Carbonyl

**HRMS** (ESI)
Composition
$C_{24}H_{26}O_8$
Calc.
$[M + H]^+$
443.17004 amu
Found
$[M + H]^+$
443.17224 amu
Delta (ppm)
4.95

**^{1}H-NMR** (400 MHz, $CDCl_3$)			
δ (ppm)	Mult. (J (Hz))	Int.	Assignment
1.49	d (7)	6	**CH_3** (L)
2.65-2.79	m	4	**CH_2** (S)
5.12-5.20	d, d, q (12.5, 12.5, 7)	6	**CH_2, CH** (Bn, L)
7.31-7.39	m	10	Aromatic (Bn)

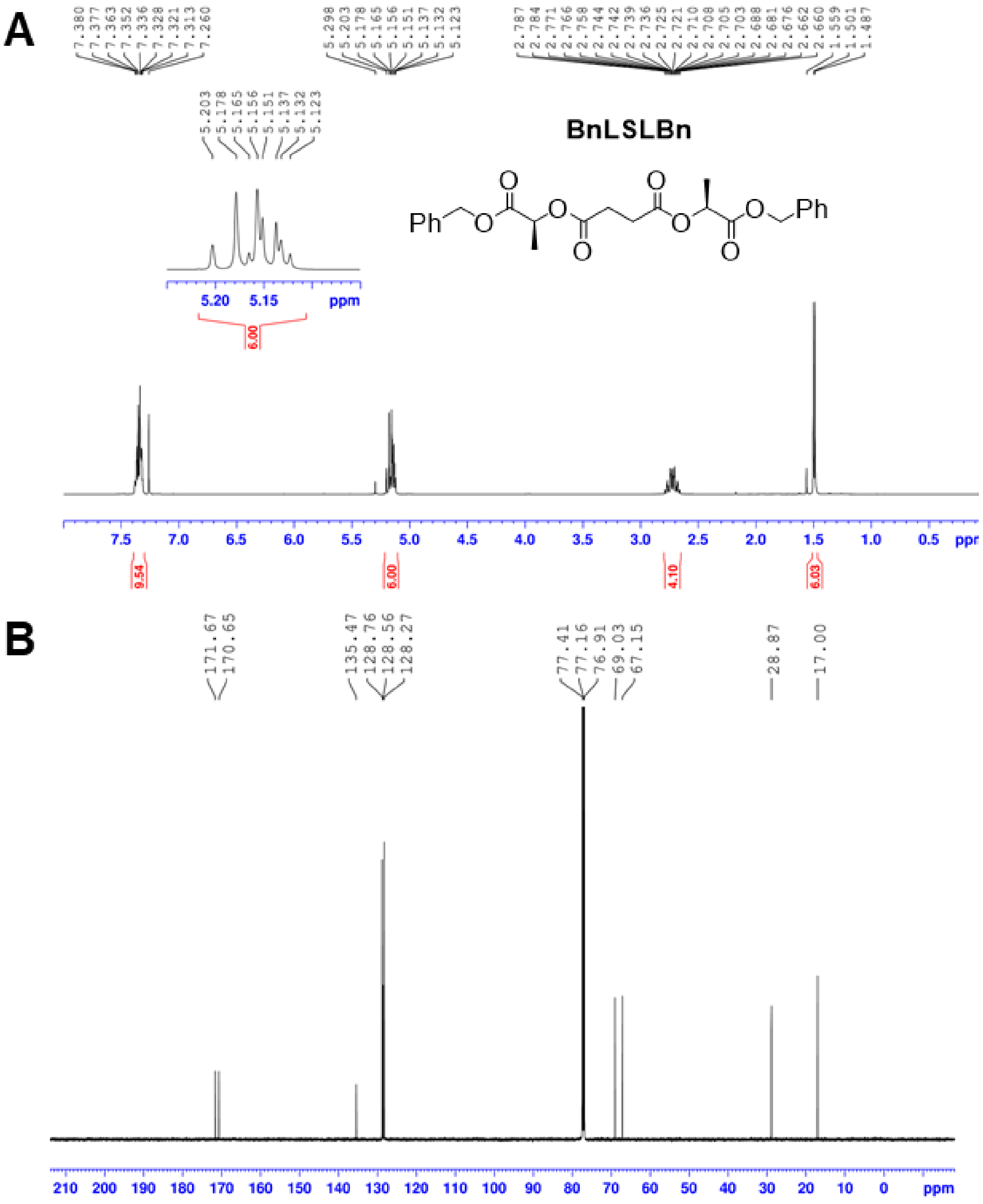

Figure S5. NMR spectra of BnLSLBn. A) ^{1}H NMR, 400 MHz, $CDCl_3$ B) ^{13}C NMR, 100 MHz, $CDCl_3$

LSL ("S" in main text). BnLSLBn (82.2 mg, 0.186 mmol) and 10% Pd/C (0.0101 g, 0.009 mmol) were added to 1.8 mL of dry EtOAc while stirring. The vial was purged with H_2 gas

three times, and a hydrogen balloon was attached to the vial. After stirring for 18 h, the reaction was filtered through celite and concentrated under reduced pressure. The resulting white solid required no further purification (44.6 mg, 91.7 %) ^{1}H NMR (400 MHz, $CDCl_3$) δ 8.0 (br, 2 H), 5.14 (q, J = 7.0 Hz, 2 H), 2.75 (m, 4 H), 1.53 (d, J = 7.0 Hz, 3 H); ^{13}C NMR (100 MHz, $CDCl_3$) δ 176.4, 171.7, 68.7, 28.8, 16.8; HRMS (ESI) calcd. mass 263.07614, found 263.07708.

LSL ("S" in main text)

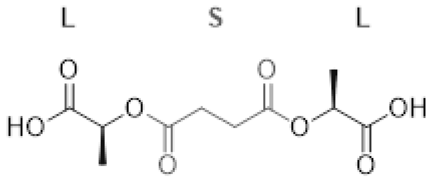

^{1}**H-NMR** (400 MHz, $CDCl_3$)			
δ (ppm)	Mult. (J (Hz))	Int.	Assignment
1.53	d (7.2)	6	C**H$_3$** (L)
2.69-2.82	m	4	C**H$_2$** (S)
5.14	q (6.8)	2	C**H** (L)
7.5-8.5	br	2	COO**H** (L)

^{13}**C-NMR** (100 MHz, $CDCl_3$)	
δ (ppm)	Assignment
16.82	**C**H_3 (L)
28.83	**C**H_2 (S)
68.65	**C**H (L)
171.74	Carbonyl
176.37	Carbonyl

**HRMS** (ESI)
Composition
$C_{10}H_{14}O_8$
Calc.
$[M - H]^-$
261.06049 amu
Found
$[M - H]^-$
261.06103 amu
Delta (ppm)
2.06

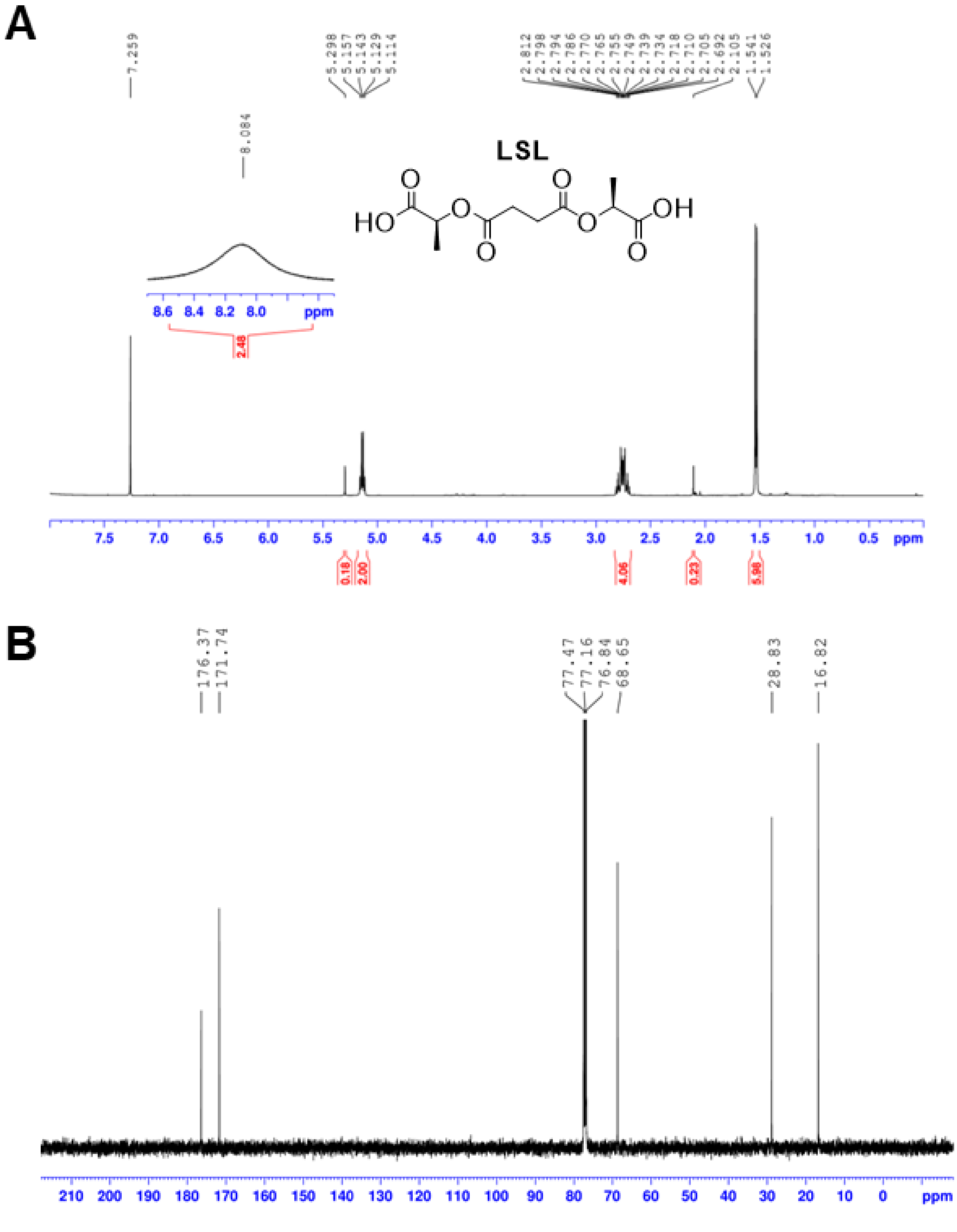

Figure S6. NMR spectra of LSL (“S” in main text). A) ^{1}H NMR, 400 MHz, $CDCl_3$ B) ^{13}C NMR, 100 MHz, $CDCl_3$

Synthesis of LUL ("U" in main text)

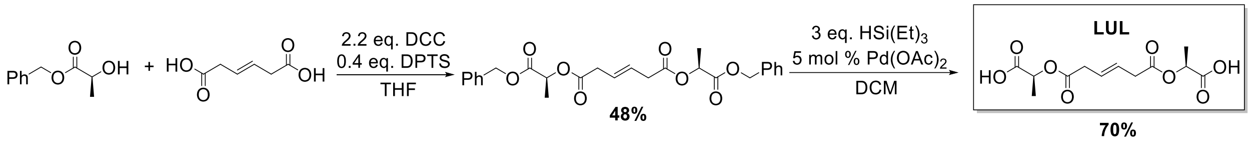

BnLULBn. Trans-β-Hydromuconic acid (1.07 g, 7.4 mmol) was added to 300 mL dry THF while stirring. The mixture was gently heated until all solid dissolved. BnL (3.0 g, 16.4 mmol) and DPTS (0.43 g, 1.4 mmol) were then added. The DPTS did not fully dissolve. DCC (3.34 g, 16.2 mmol) was then added. The reaction was tracked by TLC, and after 44 hours monosubstituted product was still visible, so additional DCC (3.7 g, 17.9 mmol) was added to push the reaction to completion. After an additional 24 h, the reaction was filtered and concentrated under reduced pressure. The crude material was purified by flash chromatography (SiO_2, 5-25% EtOAc in hexanes) to produce the pure oil (1.67 g, 48 %). ^1H NMR (500 MHz, $CDCl_3$) δ 7.35 (m, 10 H), 5.7 (m, 2H), 5.16 (m, 6 H), 3.15 (m, 4 H), 1.53 (d, J = 7.0 Hz, 6 H); ^{13}C NMR (125 MHz, $CDCl_3$) δ 171.0, 170.7, 135.5, 128.8, 128.6, 128.3, 125.9, 69.0, 67.2, 28.9, 17.0; HRMS (ESI) calcd. mass 469.18, found 443.17224.

BnLULBn

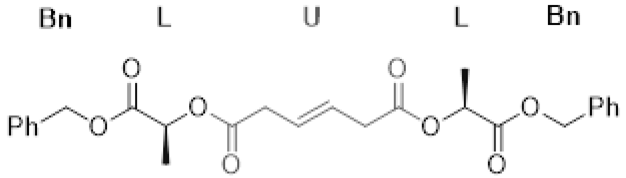

^1H-NMR (500 MHz, $CDCl_3$)

δ (ppm)	Mult. (J (Hz))	Int.	Assignment
1.50	d (7.5)	6	CH_3 (L)
3.12-3.19	m	4	CH_2 (U)
5.10-5.20	d, d, q (12, 12, 7)	6	CH_2 (Bn), CH (L)
5.68-5.71	m	2	CH (U)
7.26-7.38	m	10	Aromatic

^{13}C-NMR (125 MHz, $CDCl_3$)

δ (ppm)	Assignment
17.02	CH_3 (L)
37.55	CH_2 (U)
67.18	CH_2 (Bn)
68.98	CH (L)
125.89	CH (M)
128.28	Aromatic
128.57	Aromatic
128.76	Aromatic
135.45	Aromatic
170.67	Carbonyl
171.01	Carbonyl

HRMS (ESI)

Composition
$C_{26}H_{28}O_8$

Calc.
$[M + H]^+$
469.18569 amu

Found
$[M + H]^+$
469.18632 amu

Delta (ppm)
1.33

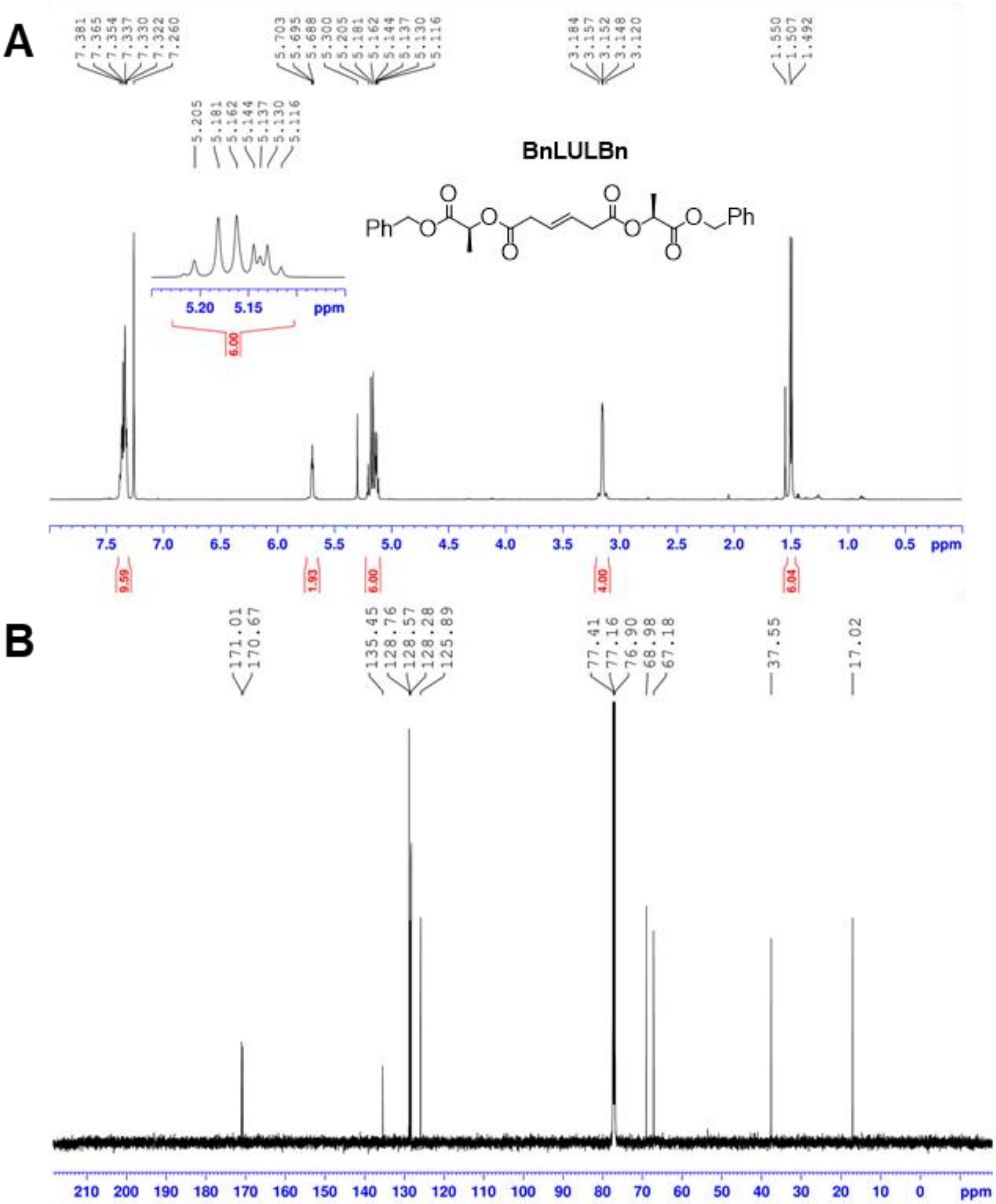

Figure S7. NMR spectra of BnLULBn. A) ^{1}H NMR, 500 MHz, $CDCl_3$ B) ^{13}C NMR, 125 MHz, $CDCl_3$

LUL (“U” in main text). Pd(OAc)$_2$ (70 mg, 0.3 mmol) was added to 30 mL dry DCM along with HSi(Et)$_3$ (3.0 mL, 19.1 mmol) and allowed to stir for 15 minutes. BnLMLBn (3.0 g, 6.4 mmol) was added along with DCM until the total volume was approximately 85 mL. After 1.5 hours, the reaction was quenched with a small amount of sat. NH_4Cl (aq). The mixture was allowed to stir for 15 minutes before drying over $MgSO_4$, filtering and concentrating under reduced pressure. The crude material was purified by flash chromatography (SiO_2, 10-50% EtOAc in hexanes) to produce the pure oil (1.29 g, 70 %). ^{1}H NMR (400 MHz, $CDCl_3$) δ 8.70 (br, 2 H), 5.14 (q, J = 7.0 Hz, 2 H), 2.75 (m, 4 H), 1.53 (d, J = 7.0 Hz, 3 H); ^{13}C NMR (100 MHz, $CDCl_3$) δ 176.4, 171.7, 68.7, 28.8, 16.8; HRMS (ESI) calcd. mass 263.07614, found 263.07708.

LUL (“U” in main text)

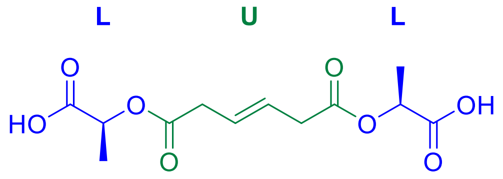

^{1}H-NMR (400 MHz, $CDCl_3$)

δ (ppm)	Mult. (J (Hz))	Int.	Assignment
1.53	d (7.5)	6	CH_3 (L)
3.13-3.23	m	4	CH_2 (U)
5.13	q (7)	2	CH (L)
5.71-5.74	m	2	CH (U)
10-10.5	br	2	COOH (L)

^{13}C-NMR (100 MHz, $CDCl_3$)

δ (ppm)	Assignment
16.85	CH_3 (L)
37.40	CH_2 (U)
68.51	CH (L)
125.86	CH (U)
171.15	Carbonyl
176.49	Carbonyl

HRMS (ESI)

Composition
$C_{12}H_{16}O_8$

Calc.
$[M + Na]^+$
311.07429 amu

Found
$[M + Na]^+$
311.07540 amu

Delta (ppm)
3.57

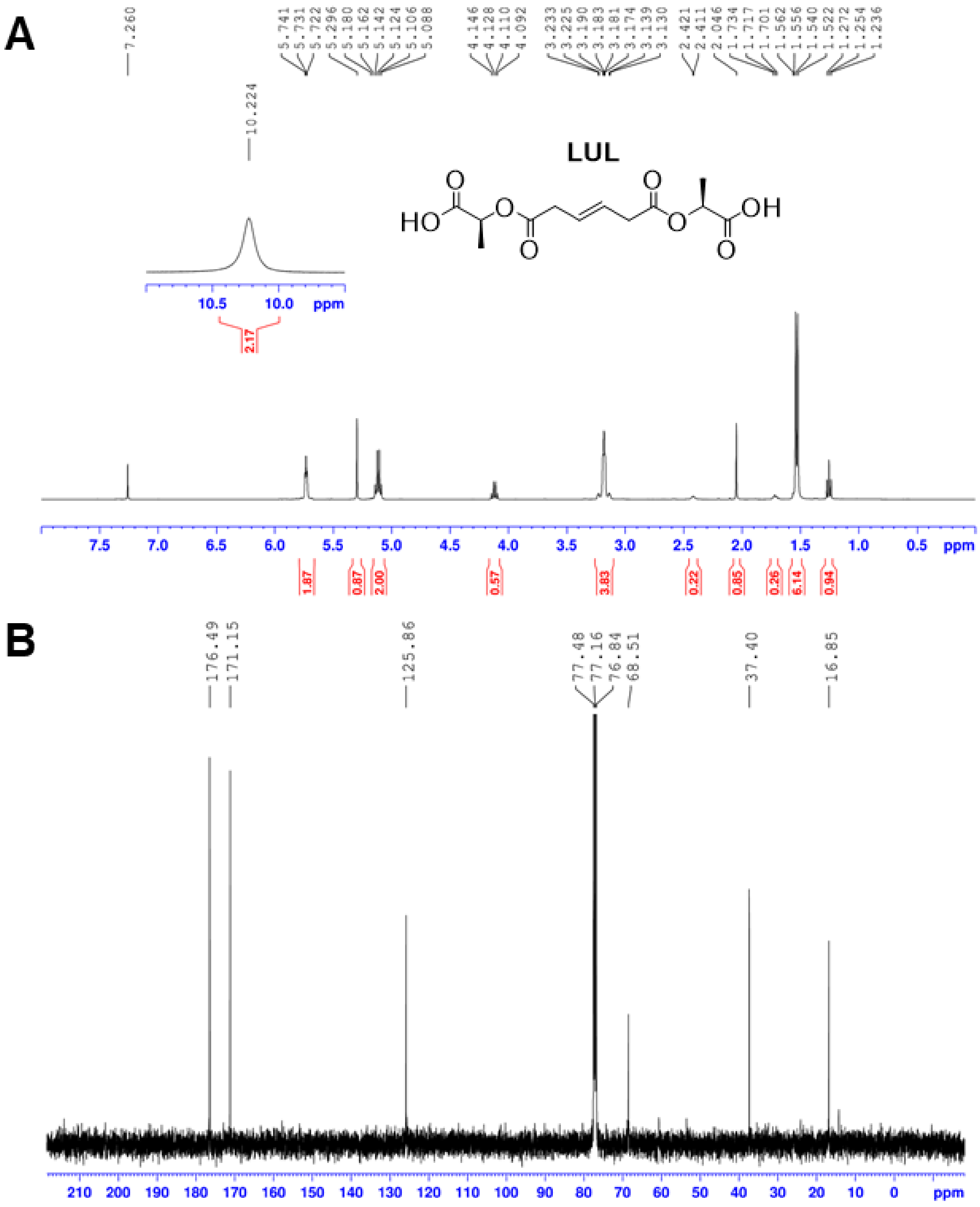

Figure S8. NMR spectra of LUL ("U" in main text). A) ^1H NMR, 500 MHz, $CDCl_3$ B) ^{13}C NMR, 125 MHz, $CDCl_3$

Polymerization General Procedure. All random polymerizations were carried out with a 1:1 mole ratio of the **G** to **U/S** units to the degree that this was possible. A typical polymer is synthesized by first combining the monomers in the appropriate ratios, then dissolving in DCM, followed by the addition of the DPTS catalyst and DIC as the coupling reagent. After 2-3 hours, the polymer is precipitated from methanol. Rand(U0) and Rand(U100) were synthesized as references to aid in characterizing other copolymers. The data for these polymers is included here for reference but was not utilized in the main text.

Rand(U0). GAG (103.8 mg, 0.47 mmol), LSL (123.5 mg, 0.47 mmol), DPTS (46.8 mg, 0.16 mmol) and DIC (162 µL, 1.03 mmol) were combined using the general polymerization procedure above. 118.8 mg polymer (52.3 %) was collected after precipitation. ^{1}H NMR (400 MHz, $CDCl_3$) δ 5.19 (q, 2 H), 4.75 (d, 2 H), 4.59 (d, 2H), 3.98 (4H, m), 2.75 (4H, m), 1.56 (6H, d), 0.97 (s, 6H); SEC (RI, THF) M_n = 8.5 kDa, M_w = 15.9 kDa, Đ = 1.9

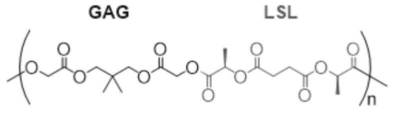

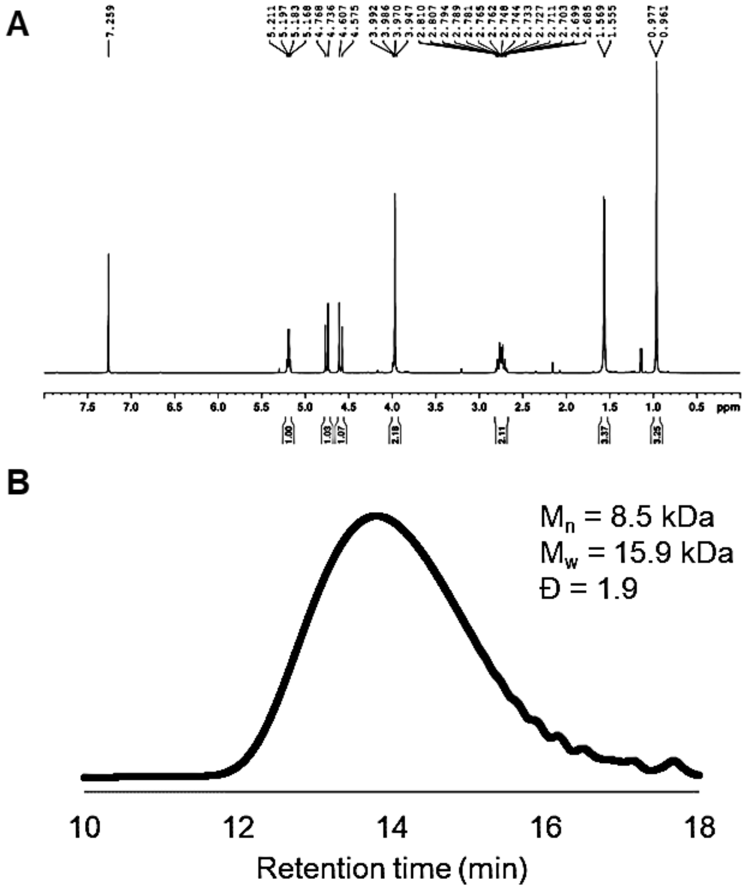

Figure S9. Characterization data for P(U0). A) ^{1}H NMR, 500 MHz, $CDCl_3$. B) SEC data vs polystyrene standards.

Rand(U100). GAG (212.7 mg, 0.97 mmol), LUL (278.4 mg, 0.97 mmol), DPTS (87.3 mg, 0.30 mmol) and DIC (334 µL, 2.13 mmol) were combined using the general polymerization procedure above. 371.8 mg polymer (75.7 %) was collected after precipitation. ^{1}H NMR (400 MHz, $CDCl_3$) δ 5.75 (m, 2H), 5.19 (q, 2 H), 4.75 (d, 2 H), 4.59 (d, 2H), 3.97 (m, 4H), 3.18 (m, 4H), 1.56 (d, 6H), 0.96 (s, 6H); SEC (RI, THF) M_n = 15.8 kDa, M_w = 35.8 kDa, Đ = 1.9

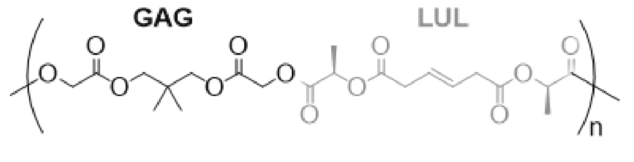

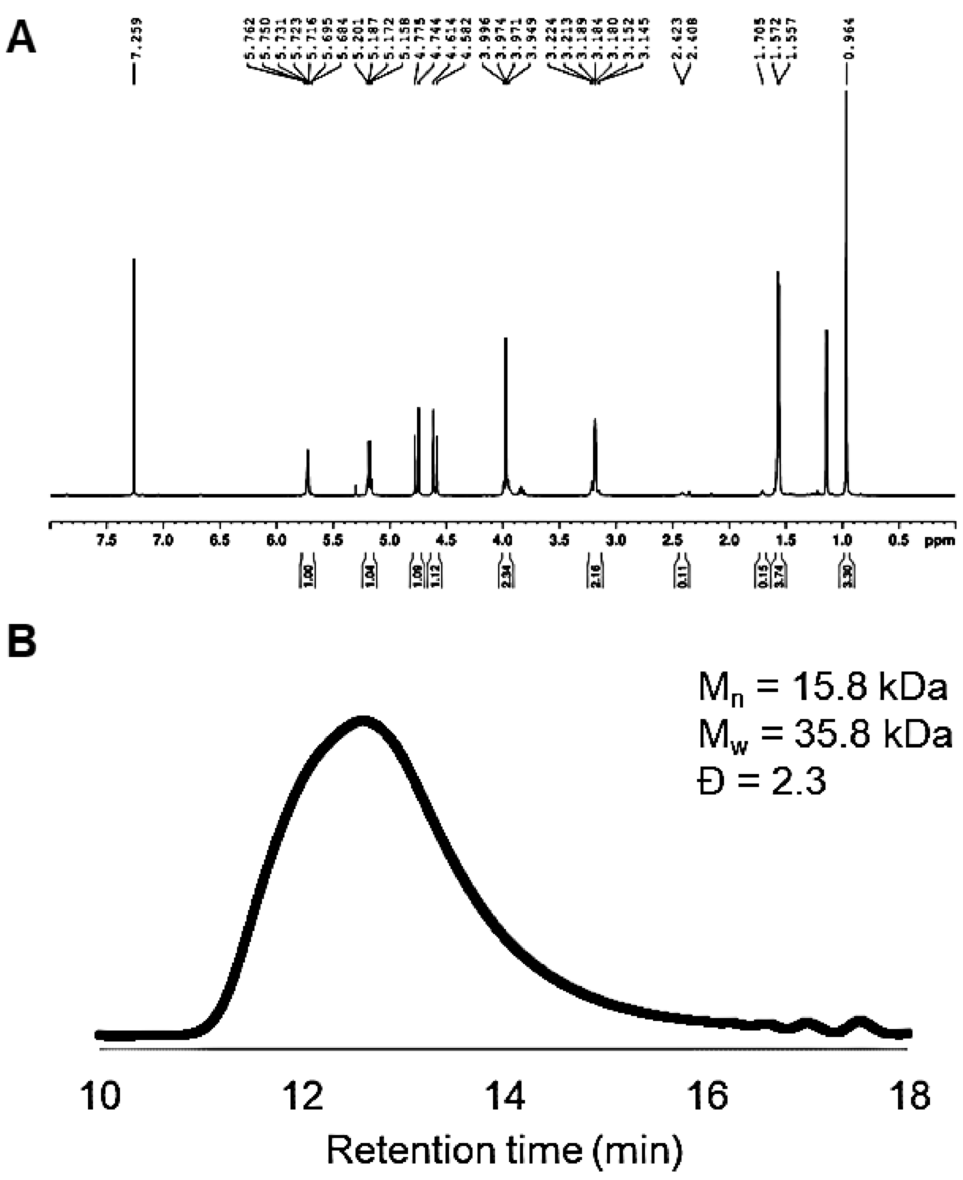

Figure S10. Characterization data for Rand(U100). A) ^{1}H NMR, 500 MHz, $CDCl_3$. B) SEC data vs polystyrene standards.

Rand(U80) example polymerization. GAG (104.2 mg, 0.47 mmol), LUL (108.8 mg, 0.38 mmol), LSL (25.0 mg, 0.01 mmol), DPTS (19.3 mg, 0.07 mmol) and DIC (200 µL, 1.20 mmol) were combined using the general polymerization procedure above. 148.2 mg polymer (62.3 %) was collected after precipitation. ^{1}H NMR (400 MHz, $CDCl_3$) δ 5.72 (m, 2H), 5.19 (q, 2 H), 4.75 (d, 2 H), 4.59 (d, 2H), 3.97 (m, 4H), 3.18 (m, 4H), 2.77 (m, 4H), 1.56 (d, 6H), 0.96 (s, 6H); SEC (RI, THF) M_n = 9.6 kDa, M_w = 13.2 kDa, Đ = 1.4

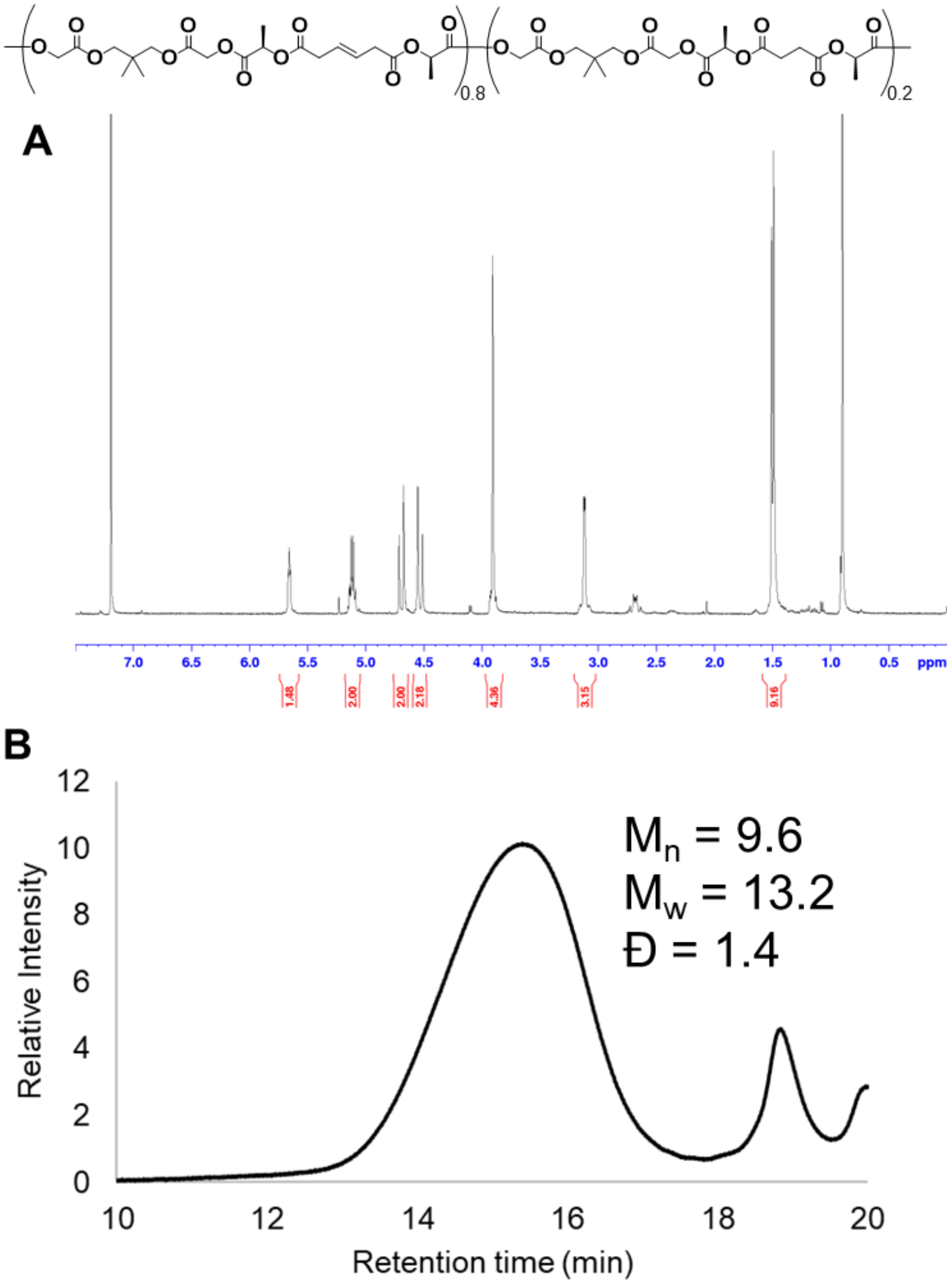

Figure S11. Characterization data for Rand(U80). A) ^{1}H NMR, 500 MHz, $CDCl_3$. B) SEC data vs polystyrene standards.

Rand(U20) example polymerization. GAG (104.1 mg, 0.47 mmol), LUL (28.4 mg, 0.01 mmol), LSL (98.1 mg, 0.37 mmol), DPTS (19.3 mg, 0.07 mmol) and DIC (200 µL, 1.28 mmol) were combined using the general polymerization procedure above. 167.0 mg polymer (72.4 %) was collected after precipitation. ^{1}H NMR (400 MHz, $CDCl_3$) δ 5.72 (m, 2H), 5.19 (q, 2 H), 4.75 (d, 2 H), 4.60 (d, 2H), 3.97 (m, 4H), 3.19 (m, 4H), 2.77 (m, 4H), 1.56 (d, 6H), 0.96 (s, 6H); SEC (RI, THF) M_n = 9.2 kDa, M_w = 13.2 kDa, Đ = 1.4

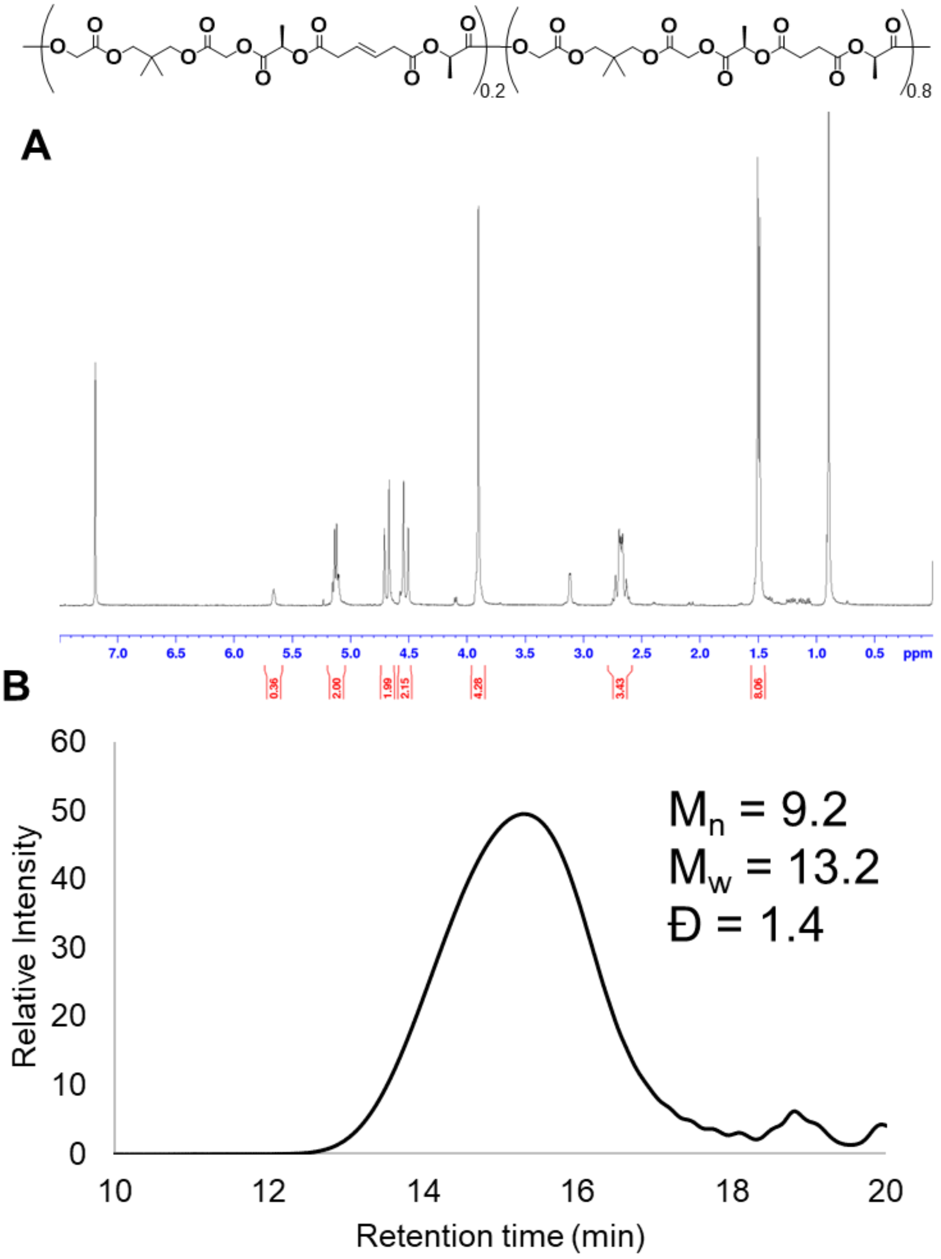

Figure S12. Characterization data for Rand(U20). A) ^{1}H NMR, 500 MHz, $CDCl_3$. B) SEC data vs polystyrene standards.

Parallel-Successive Copolymerization General Procedure.

$(U60)_5$ diacid: GAG (19.7 mg, 0.090 mmol, 1 eq.) was combined with LSL (15.6 mg, 0.06 mmol, 0.66 eq.), LUL (21.5 mg, 0.075 mmol, 0.83 eq.), and DPTS (3.0 mg, 0.01 mmol) in 3.0 mL dry DCM while stirring. DIC (32 uL, 0.2 mmol) was added and the reaction was allowed to stir for 2 hours. SEC-RI: Mn = 1.2 kDa, Đ = 1.1.

$(U0)_{10}$ diol: GAG (59.0 mg, 0.268 mmol, 1 eq.) was combined with LSL (58.5 mg, 0.22 mmol, 0.83 eq.), and DPTS (10.0 mg, 0.03 mmol) in 3.0 mL dry DCM while stirring. DIC (95 uL, 0.6 mmol) was added and the reaction was allowed to stir for 2 hours. SEC-RI: Mn = 2.4 kDa, Đ = 1.7.

$(U60)_5$-*alt*-$(U0)_{10}$: After two hours of reaction time, ~50 uL of each oligomerization reaction were removed for SEC analysis. Each oligomerization reaction was then gently concentrated by evaporation under active nitrogen until each had a volume of approximately 1 mL. The **$(U60)_5$** diacid was then transferred to the **$(U0)_{10}$** reaction with DCM. After the transfer was completed, DPTS (3.0 mg, 0.01 mmol) and DIC (17 uL, 0.1 mmol) were added and the combined reactions were allowed to stir overnight (18 h). The polymer was precipitated in methanol. SEC-RI: Mn = 7.4 kDa, Đ = 1.4.

$(U60)_5$ diacid

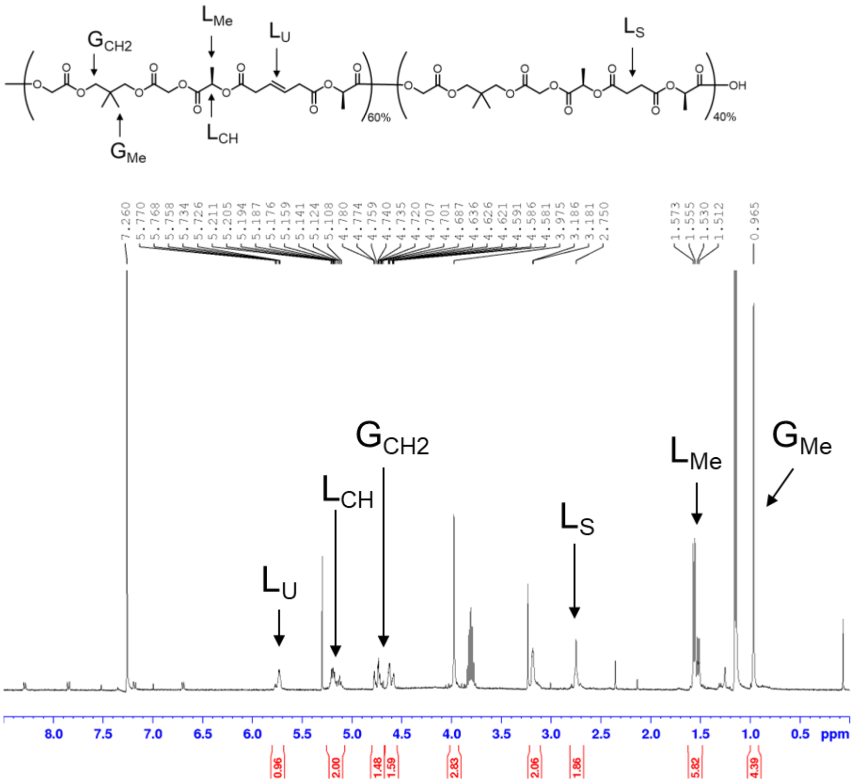

Figure S13. ^{1}H NMR data for the DP5 (60% U) diacid oligomer used to synthesize **(U60)$_5$-*alt*-(U0)$_{10}$**.

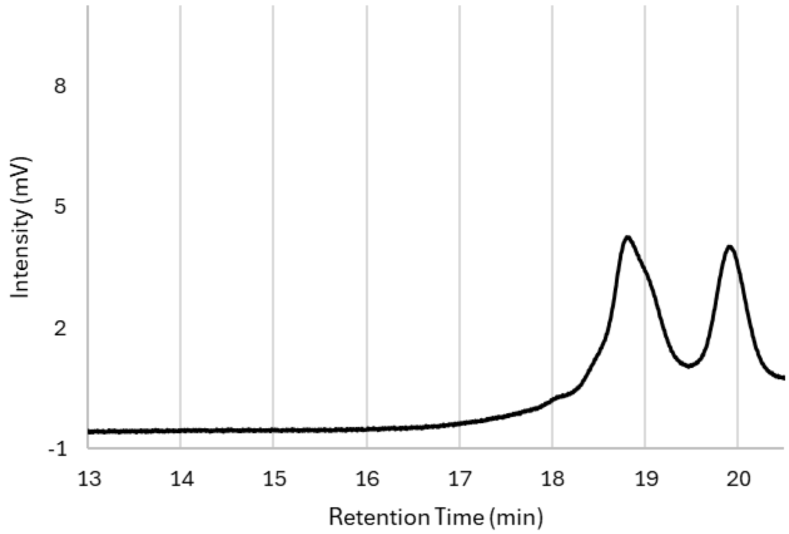

Figure S14. SEC data for the DP5 (60% U) diacid oligomer used to synthesize **(U60)$_5$-alt-(U0)$_{10}$**. SEC-RI: M_n = 1.2 kDa, Đ = 1.1. The first peak at 18.8 minutes is the oligomer, the second peak at 20 minutes is an SEC system peak.

(U0)$_{10}$ diol.

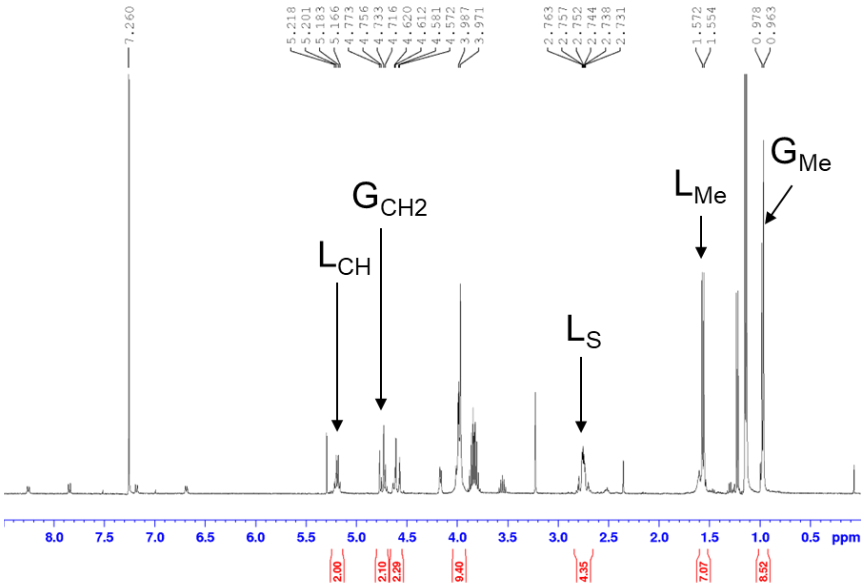

Figure S15. ^{1}H NMR data for the DP10 (0% U) diol oligomer used to synthesize **(U60)$_5$-*alt*-(U0)$_{10}$**.

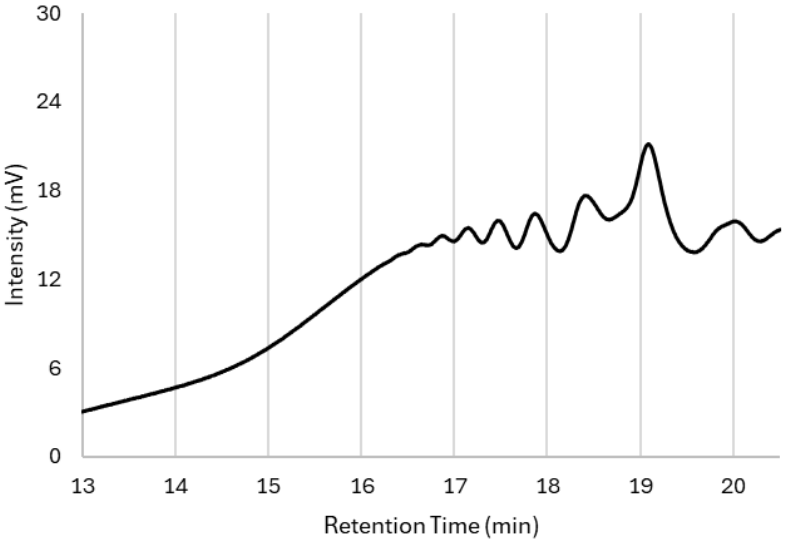

Figure S16. SEC data for the DP10 (0% U) diol oligomer used to synthesize **(U60)₅-alt-(U0)₁₀**. SEC-RI: M_n = 2.4 kDa, Đ = 1.7.

(U60)₅-*alt*-(U0)₁₀ P-S copolymer.

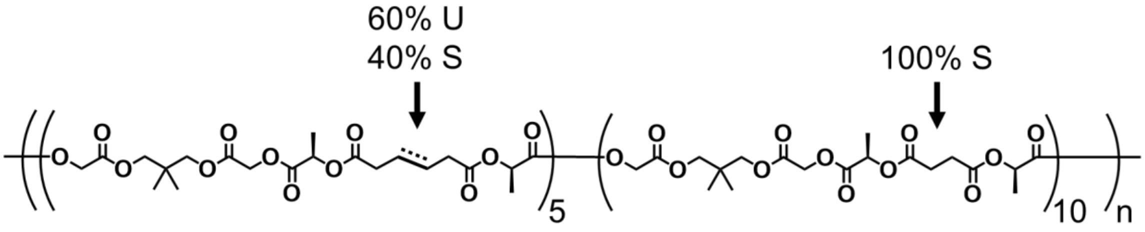

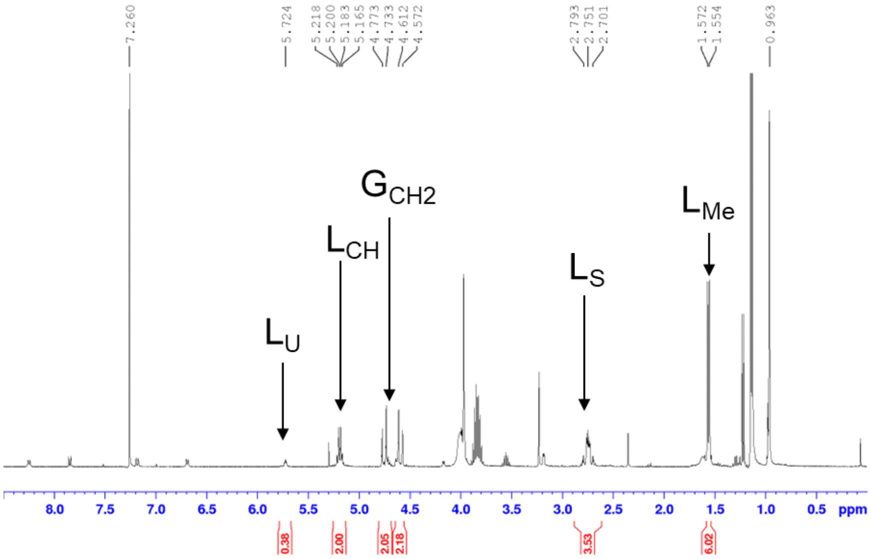

Figure S17. ^{1}H NMR data for **(U60)₅-*alt*-(U0)₁₀**.

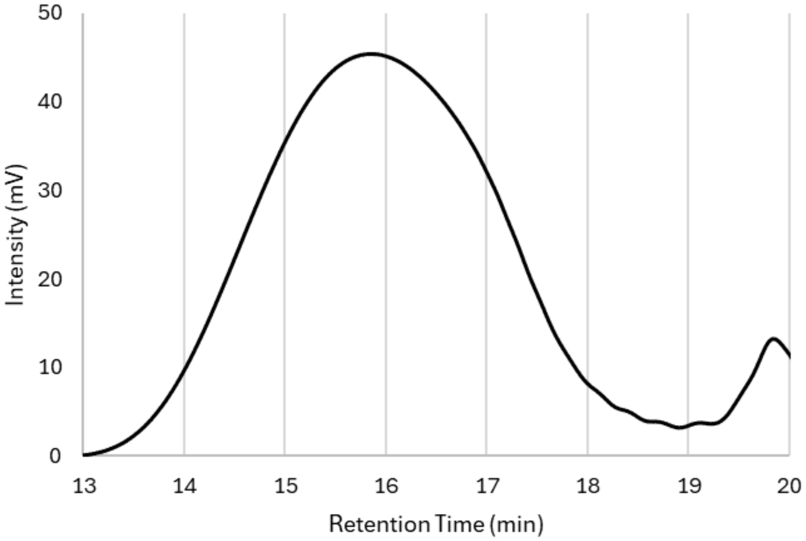

Figure S18. SEC data for **(U60)$_5$-alt-(U0)$_{10}$**. SEC-RI: M_n = 7.8 kDa, Đ = 1.4.

Cross Metathesis (CM) Digestion General Procedure. Digestions were carried out by combining the polymer with styrene and Grubb's 2nd generation catalyst (10 eq. and 5 mol % relative to the LUL content of the polymer respectively) in dry DCE. The reactions were run at 50 °C for 24 h under passive N_2 while stirring before being quenched with ethyl vinyl ether. Digestion products were filtered through celite and concentrated to dryness. The digestion products were then rinsed with hexanes to remove the majority of residual styrene dimer (stilbene) impurities. The hexanes washes were concentrated and characterized by NMR and were confirmed to only contain stilbene and none of the desired digestion products.

CM of Rand(U20) with styrene. Rand(U20) (91.7 mg), Grubb's 2nd generation catalyst (4.5 mg, 0.0053 mmol), and styrene (200 µL, 0.63 mmol) were combined using the general procedure outlined above. SEC and select ^{1}H NMR data are shown below.

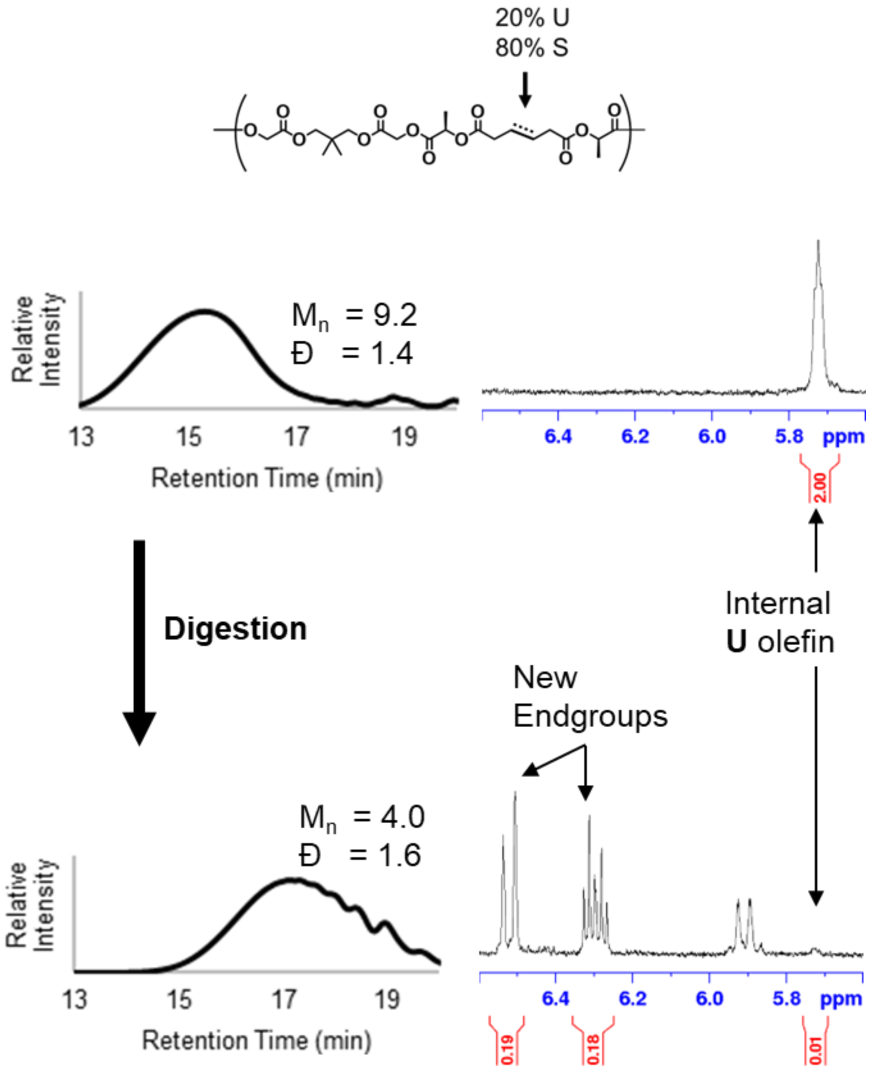

Figure S19. Characterization data for the digestion of Rand(U20). The top panel shows the SEC and ^{1}H NMR data for the pre-digested polymer. The bottom panel shows SEC and ^{1}H NMR data for the post-digestion products. Note that >95% of the internal **U** olefins were fully digested by NMR.

CM of (U60)$_5$-*alt*-(U0)$_{10}$. (U60)$_5$-*alt*-(U0)$_{10}$ (71.3 mg), Grubb's 2nd generation catalyst (4.6 mg, 0.0054 mmol), and styrene (200 µL, 0.63 mmol) were combined using the general procedure outlined above. SEC and select ^{1}H NMR data are shown below.

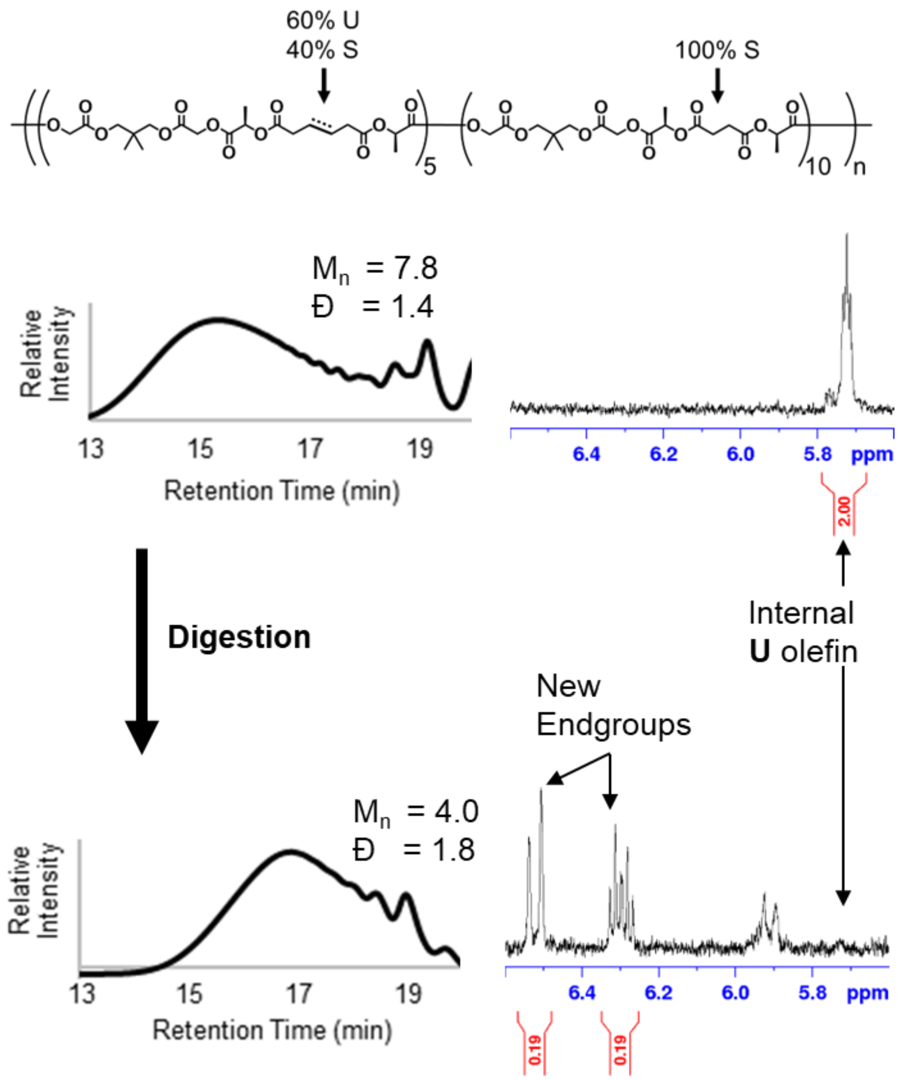

Figure S20. Characterization data for the digestion of **(U60)$_5$-*alt*-(U0)$_{10}$**. The top panel shows the SEC and 1H NMR data for the pre-digested polymer. The bottom panel shows SEC and 1H NMR data for the post-digestion products. Note that >95% of the internal **U** olefins were fully digested by NMR.

Monte Carlo Simulations.

Overview of the Polymer Generation Algorithms

The PSpolymer(DPavg1, pU1, DPavg2, pU2, NumPolymers) function simulates a parallel-successive step-growth copolymerization reaction between two sets of oligomers terminated by stoichiometric imbalance and saves the resulting polymer data. Users specify the desired average degree of polymerization (DP) and the fraction of "U" monomer content (pU) for both the diol (Set 1) and diacid (Set 2) oligomer sets. The PSpolymerDiscrete((DP1, pU1, DP2, pU2, NumPolymers) function does the exact same thing, but instead uses oligomers with defined, discrete DPs instead of oligomer pools with disperse Flory distributions.

The following section details the PSpolymer function. A table is provided to list the specific input parameters used to simulate all the copolymer variations discussed in the main text. This is followed by the fully commented source code used to generate the sequence data for both the discrete and disperse P-S copolymers.

The algorithm executes in two main phases: *Oligomer Generation* and *Final Polymer Building*.

Phase 1: Generating the Initial Oligomers

The first phase generates lists representing the starting diol and diacid oligomer pools:

1. *Determine DP Distribution:* The program calculates the necessary "r" value (stoichiometric ratio) to achieve the target average DP for each set. It then uses the Flory distribution, which accounts for stoichiometric imbalance, to determine the relative amounts of different chain lengths. The version of the Flory distribution that is used in this step assumes only odd-valued DPs are present due to capping endgroups with excess monomer. The selected distribution is normalized, scaled to the total "NumPolymers" requested, and rounded to the nearest integer count for each chain length.
2. *Assign Monomer Sequences:* Each individual chain is assigned a specific sequence of "S" and "U" monomers, ensuring the sequences are consistent with the user's input %U values (pU1 and pU2). This involves:
   - Calculating the total number of S and U monomers needed for all chains of a given DP.
   - Creating a long string of these monomers, randomizing the order.
   - Splitting the string into individual, randomized sequences of the correct length.

   (Note: The alternating "g" and capping of monomers are accounted for in a final step, meaning a diol chain with a DP of 11 needs 5 internal S/U monomers.)

Phase 2: Building the Final P-S Copolymers

The second phase combines the generated oligomers into the final, longer P-S copolymers:

1. *Combine and Alternate Lists:* The two generated lists ("Oligomer Set 1" and "Oligomer Set 2") are randomized separately. They are then merged into a single "master list" where diols and diacids strictly alternate (e.g., diol, diacid, diol, diacid...).
2. **Generate Final DP Distribution:** The oligomers are now treated as "macromonomers" reacting in an AA/BB step-growth fashion. The final resulting polymers are expected to follow the standard Flory distribution. A new Flory distribution is generated for the final polymers (this time assuming stoichiometric balance between the oligomer sets) and scaled up to integer chain counts. Both even and odd "DP"s are allowed at this stage. Here, DP refers to the number of oligomers per polymer chain.
3. **Assemble Final Chains:** Instead of using single monomers, the algorithm uses the oligomer sequences generated in Phase 1 as building blocks. The master list of alternating oligomers is split according to the final Flory distribution to construct the complete P-S copolymer dataset. When joining oligomer chains to form a polymer, a '-' is inserted between each chain, which must be later removed when extracting DP distribution data. The '-' is used to track and count the number of oligomers per polymer chain in the final dataset.

Polymer Generation Function Inputs

The following tables are the exact parameters used to generate the simulation data for the main text using the source code provided below. Each simulation took approximately ~4 hours to complete and generated 1,000,000 polymer chains per dataset. Note that smaller datasets can be generated much more quickly.

Disperse P-S Copolymers (`PSpolymer(DPavg1, pU1, DPavg2, pU2, NumPolymers)`)

Polymer Name	DPavg1	pU1	DPavg2	pU2	NumPolymers
$(U50)_5$-*alt*-$(U0)_{10}$	10	0	5	0.50	1000000
$(U47)_5$-*alt*-$(U2)_{10}$	10	0.02	5	0.47	1000000
$(U44)_5$-*alt*-$(U4)_{10}$	10	0.04	5	0.44	1000000
$(U41)_5$-*alt*-$(U6)_{10}$	10	0.06	5	0.41	1000000
$(U35)_5$-*alt*-$(U10)_{10}$	10	0.10	5	0.35	1000000
$(U29)_5$-*alt*-$(U14)_{10}$	10	0.14	5	0.29	1000000
$(U20)_5$-*alt*-$(U20)_{10}$	10	0.20	5	0.20	1000000
$(U14)_5$-*alt*-$(U24)_{10}$	10	0.24	5	0.14	1000000
$(U8)_5$-*alt*-$(U28)_{10}$	10	0.28	5	0.08	1000000
$(U5)_5$-*alt*-$(U30)_{10}$	10	0.30	5	0.05	1000000
$(U2)_5$-*alt*-$(U32)_{10}$	10	0.32	5	0.02	1000000
$(U0)_5$-*alt*-$(U33)_{10}$	10	0.3333333	5	0	1000000

Discrete P-S Copolymers (`PSpolymerDiscrete(DP1, pU1, DP2, pU2, NumPolymers)`)

Polymer Name	DP1	pU1	DP2	pU2	NumPolymers
$(U53)_5$-*alt*-$(U0)_{11}$	11	0	5	0.53333	1000000
$(U47)_5$-*alt*-$(U4)_{11}$	11	0.04	5	0.46666	1000000
$(U40)_5$-*alt*-$(U8)_{11}$	11	0.08	5	0.40	1000000
$(U33)_5$-*alt*-$(U12)_{11}$	11	0.12	5	0.33333	1000000
$(U27)_5$-*alt*-$(U16)_{11}$	11	0.16	5	0.26666	1000000
$(U20)_5$-*alt*-$(U20)_{11}$	11	0.20	5	0.20	1000000
$(U13)_5$-*alt*-$(U24)_{11}$	11	0.24	5	0.13333	1000000
$(U7)_5$-*alt*-$(U28)_{11}$	11	0.28	5	0.06666	1000000
$(U3)_5$-*alt*-$(U30)_{11}$	11	0.30	5	0.03333	1000000
$(U0)_5$-*alt*-$(U32)_{11}$	11	0.32	5	0	1000000

Polymer Generation Source Code

Disperse Oligomers

```
import random
import re
from decimal import Decimal
from collections import Counter
import pickle
import itertools

#path is the directory where data will be saved
path = ‘’

def PSpolymer(DPavg1, pU1, DPavg2, pU2, NumPolymers):
    #DPavg is the average DP of each oligomer set, where DP is number of monomers
    #pU is the fraction LUL (out of the total LUL+LSL).  Must be between 0 and 1

    #Oligomer set 1 is the GAG terminated diol (DPavg1, pU1)
    #Oligomer set 2 is the LUL/LSL terminated diacid (DPavg2, pU2)

#The current simulation assumes 90% endgroup consumption during the successive
#coupling of oligomer sets 1 and 2
    #This is currently hardcoded into the program.
    #NumPolymers is the total number of polymer chains expected in the dataset after
    #the program is finished completing
    #At 90% conversion, NumPolymers must be multiplied by 5 to obtain the correct
    #"ScaleUp" factor which will result
    #in the desired dataset size
    ScaleUp = NumPolymers*5

    #Converts input values into strings for later use in filenames
    DPoli1 = str(DPavg1)
    pUoli1 = str(int(round(Decimal(pU1*100),0)))
    DPoli2 = str(DPavg2)
    pUoli2 = str(int(round(Decimal(pU2*100),0)))

    #Calculates stoichiometric imbalance ratio for Flory distribution based on the
    #desired average DP
    #Carothers equation
    r1 = (DPavg1-1)/(DPavg1+1)

    #Empty lists for DP and probabilities
    DPlist1 = []
    Problist1 = []

#Flory distribution for stoichiometric imbalance reflects the number of A-A and
#B-B monomers per chain,
#At equillibrium under stoichiometric imbalance conditions, all chains are capped
#and have an odd number of monomers.
    #The initial for-loop generates a list of odd values from 1 to 1999.
    #These are the number of monomers (A-A and B-B) in a given oligomer/polymer chain.
    for n1 in range(1,1999):
        if n1 % 2 == 0:
            pass
    #The following calculates the probability of finding a chain of length "n1" at the
    #calculated "r1"
    #n1 is the number of monomers in the chain, and r1 is the stiochiometric imbalance
    #ratio calculated above
    #the probability value will dictate the number of times a chain of length n1 is generated.
    #The factor "ScaleUp"
    #increases the number of oligomer chains ultimately generated, but has no effect
    #on the distribution pattern.
        else:
```

```
        probability = r1**(n1/2)*(1-r1)*r1**(-1/2)*ScaleUp
#In order for the simulation to generate the correct number of oligomer chains,
#the probability value must be converted to an integer
        probability = int(round(Decimal(probability),0))
        dp = n1
#This last piece adds all non-zero probabilities and DPs to the final lists that
#will be used to generate the oligomer data
        if probability > 0:
            DPlist1.append(dp)
            Problist1.append(probability)
NumChains1 = sum(Problist1)

#The entire process above is repeated for oligomer set 2
r2 = (DPavg2-1)/(DPavg2+1)
DPlist2 = []
Problist2 = []

for n2 in range(1,1999):
    if n2 % 2 == 0:
        pass
    else:
        probability = r2**(n2/2)*(1-r2)*r2**(-1/2)*ScaleUp
        probability = int(round(Decimal(probability),0))
        dp = int(n2)
        if probability > 0:
            DPlist2.append(dp)
            Problist2.append(probability)
NumChains2 = sum(Problist2)

Olig1 = dict(zip(DPlist1,Problist1))
Olig2 = dict(zip(DPlist2,Problist2))

#The following generates the oligomer sequences for oligomer set 1
#pU1 is the feed ratio percentage of 'U' and pS1 is the feed ratio percentage of 'S'
pS1 = 1-pU1
OligSeq1 = []
#This code generates a random U/S sequence for every oligomer in the data set.
#The length of the oligomer chain and
#the number of chains is dictated by the flory distribution calculated earlier.
#The feed ratios of U and S are
#dictated by the simulation inputs.

#Oligomer set one is the 'g' diol terminated set
#Since the diol (g) is in excess, any length 1 chains must be 'g'
for monomer in range(Problist1[0]):
    OligSeq1.append('g')
#'seq' creates a string of U's and S's that represent the number of monomers
#in all chains for a specific DP
#For example, if there a 100 DP=5 oligomers with 50%U and 50% S feed ratios,
#'seq' will create a string that
#contains at least 250 'u's and 250 's's in a single string. Not all characters
#in 'seq' may need to be used.
#'length' creates a string of '.'s equal to the DP of the oligomer.
#So for DP = 5, length = '.....'
#'seqlist' first randomizes the list of 'u's and 's's in 'seq', and then
#divides the entire list into chunks
#of length 5, and collects as many oligomers as are needed to fullfil the
#Flory Distribution requirements
#The final for-loop simply adds the endgroups ('g') and alternates g appropriately
#between the 'u's and 's's.
for DP in range(1,len(Problist1)):
    seq = 'u'*int((DP)*pU1*Problist1[DP])+'s'*int((DP)*pS1*Problist1[DP])
    length = '.'*(DP)
    seqlist = ''.join(random.sample(seq, len(seq)))
    seqlist = re.findall(length,seqlist)
```

```
        for i in range(len(seqlist)):
            seqlist[i] = 'g'+'g'.join(seqlist[i])+'g'
        OligSeq1.extend(seqlist)

    #Oligomer set two is the 'u' or 's' diacid terminated set. Since the diacid is
    #in excess, it is simply handled by the function below. Notice the counting in
    #the for loop starts at 0 this time. Because of this, we need to express the DP as
    #n+1 for the math to work out. Also, we do not need to add 'g' endgroups, but we
    #still need to add the alternating g monomers.

    pS2 = 1-pU2
    OligSeq2 = []
    for n in range(0,len(Problist2)):
        DP = n+1
        seq = 'u'*int((DP)*pU2*Problist2[n])+'s'*int((DP)*pS2*Problist2[n])
        length = '.'*(DP)
        seqlist = ''.join(random.sample(seq, len(seq)))
        seqlist = re.findall(length,seqlist)
        for i in range(len(seqlist)):
            seqlist[i] = 'g'.join(seqlist[i])
        OligSeq2.extend(seqlist)

    #renaming variables for oligomer set lists
    Oli1, Oli2 = OligSeq1, OligSeq2

    #intermediate output files for the oligomer datasets for testing purposes
    filename1 = path+'Oligs\\DP'+DPoli1+'-'+DPoli2+'_pU'+pUoli1+'-'+pUoli2+'_Oli1'
    filename2 = path+'Oligs\\DP'+DPoli1+'-'+DPoli2+'_pU'+pUoli1+'-'+pUoli2+'_Oli2'

    with open(filename1, 'wb') as out:
        pickle.dump(Oli1, out)

    with open(filename2, 'wb') as out:
        pickle.dump(Oli2, out)

    #Randomizes order of oligomer datasets
    Olig1 = random.sample(Oli1,k=len(Oli1))
    Olig2 = random.sample(Oli2,k=len(Oli2))

    #Oligomer dataset sizes are different by 50-100 chains (random). Each Olig
    #dataset contains ~7.5 million chains.
    #The following makes sure the datasets are the same size by truncating the smaller dataset
    if len(Olig2) > len(Olig1):
        Olig2 = Olig2[:len(Olig1)]
    elif len(Olig1) > len(Olig2):
        Olig1 = Olig1[:len(Olig2)]
    else:
        pass

    #This combines the oligomer pools into a single list of alternating chains
    Oligs = list(zip(Olig2, Olig1))
    OligPool = list(itertools.chain.from_iterable(Oligs))


    #This generates the overall Flory distribution of the final copolymer.
    #Here "DP" indicates the number of oligomers per chain.
    #p = extent of reaction
    #n = DP
    totalOligs = len(OligPool)
    p = 0.9
    avgdp = 1/(1-p)

    OligDPlist = []
    OligProblist = []

```

```
    for n in range(1,1000):
        probability = (1-p)*p**(n-1)*totalOligs/avgdp
        probability = int(round(Decimal(probability),0))
        if probability > 0:
            OligDPlist.append(n)
            OligProblist.append(probability)

    #The following for loop builds the final copolymers.
    #The Flory distribution dictates the number of chains (x) for a given DP (n)
    #The Oligomers are pulled from the altnernating master list of oligomers
    Polymers = []
    for n in range(0,len(OligProblist)):
        for x in range(0,OligProblist[n]):
            chains = []
            DP = n+1
            chains = OligPool[:DP]
            del OligPool[:DP]
            chain = "-".join(chains)
            Polymers.append(chain)
    Polymers.extend(OligPool)

    #Saves and outputs the data
    filename3 = path+'Poly\\DP'+DPoli1+'-'+DPoli2+'_pU'+pUoli1+'-'+pUoli2+'_Poly'
    with open(filename3, 'wb') as out:
        pickle.dump(Polymers, out)

    return Polymers

```

Discrete Oligomers

```
import random
import re
from decimal import Decimal
from collections import Counter
import pickle
import itertools
#path is the directory where data will be saved
path = ‘’

def PSpolymerDiscrete(DP1, pU1, DP2, pU2, NumPolymers):
    #DP is number of monomers per oligomer. Must be an odd integer value
    #pU is the fraction LUL (out of the total LUL+LSL).  Must be between 0 and 1
    #Oligomer set 1 is the GAG terminated diol (DPavg1, pU1)
    #Oligomer set 2 is the LUL/LSL terminated diacid (DPavg2, pU2)

    #The current simulation assumes 90% endgroup consumption during the
    #successive coupling of oligomer sets 1 and 2
    #This is currently hardcoded into the program.
    #NumPolymers is the total number of polymer chains expected in the dataset
    #after the program is finished completing
    #At 90% conversion, NumPolymers must be multiplied by 5 to obtain the
    #correct "ScaleUp" factor which will resultin the desired dataset size

    ScaleUp = NumPolymers*5

    #Converts input values into strings for later use in filenames
    DPoli1 = str(DP1)
    pUoli1 = str(int(round(Decimal(pU1*100),0)))
    DPoli2 = str(DP2)
    pUoli2 = str(int(round(Decimal(pU2*100),0)))

    #generates the discrete oligomers based on the input DP and desired U
    #composition. Olig 1 is the g terminated diol. For a chain of length DP,
    #it will have (DP-1)/2 diacid monomers
    pS1 = 1-pU1
    OligSeq1 = []
    usDP1 = int(round(Decimal((DP1-1)/2),0))
    seq = 'u'*int(usDP1*pU1*ScaleUp)+'s'*int(usDP1*pS1*ScaleUp)
    length = '.'*(usDP1)
    seqlist = ''.join(random.sample(seq, len(seq)))
    seqlist = re.findall(length,seqlist)
    for i in range(len(seqlist)):
        seqlist[i] = 'g'+'g'.join(seqlist[i])+'g'
    OligSeq1.extend(seqlist)

    #Olig 2 is the u/s terminated diacid. For a chain of length DP,
    #it will have (DP+1)/2 diacid monomers
    pS2 = 1-pU2
    OligSeq2 = []
    usDP2 = int(round(Decimal((DP2+1)/2)))
    seq = 'u'*int(usDP2*pU2*ScaleUp)+'s'*int(usDP2*pS2*ScaleUp)
    length = '.'*(usDP2)
    seqlist = ''.join(random.sample(seq, len(seq)))
    seqlist = re.findall(length,seqlist)
    for i in range(len(seqlist)):
        seqlist[i] = 'g'.join(seqlist[i])
    OligSeq2.extend(seqlist)

    #renaming variables for oligomer set lists
    Oli1, Oli2 = OligSeq1, OligSeq2

    #intermediate output files for the oligomer datasets for testing purposes
```

```
    filename1 = path+'Oligs\\DP'+DPoli1+'-'+DPoli2+'_pU'+pUoli1+'-'+pUoli2+'_Oli1'
    filename2 = path+'Oligs\\DP'+DPoli1+'-'+DPoli2+'_pU'+pUoli1+'-'+pUoli2+'_Oli2'

    with open(filename1, 'wb') as out:
        pickle.dump(Oli1, out)

    with open(filename2, 'wb') as out:
        pickle.dump(Oli2, out)

    #Randomizes order of oligomer datasets
    Olig1 = random.sample(Oli1,k=len(Oli1))
    Olig2 = random.sample(Oli2,k=len(Oli2))

    #Oligomer dataset sizes are different by 50-100 chains (random).
    #Each Olig dataset contains ~7.5 million chains.
    #The following makes sure the datasets are the same size by truncating
    #the smaller dataset
    if len(Olig2) > len(Olig1):
        Olig2 = Olig2[:len(Olig1)]
    elif len(Olig1) > len(Olig2):
        Olig1 = Olig1[:len(Olig2)]
    else:
        pass

    #This combines the oligomer pools into a single list of alternating chains
    Oligs = list(zip(Olig2, Olig1))
    OligPool = list(itertools.chain.from_iterable(Oligs))

    #This generates the overall Flory distribution of the final copolymer.
    #Here "DP" indicates the number of oligomers per chain.
    #p = extent of reaction
    #n = DP
    totalOligs = len(OligPool)
    p = 0.9
    avgdp = 1/(1-p)
    OligDPlist = []
    OligProblist = []

    for n in range(1,1000):
        probability = (1-p)*p**(n-1)*totalOligs/avgdp
        probability = int(round(Decimal(probability),0))
        if probability > 0:
            OligDPlist.append(n)
            OligProblist.append(probability)

    #The following for loop builds the final copolymers.
    #The Flory distribution dictates the number of chains (x) for a given DP (n)
    #The Oligomers are pulled from the altnernating master list of oligomers
    Polymers = []
    for n in range(0,len(OligProblist)):
        for x in range(0,OligProblist[n]):
            chains = []
            DP = n+1
            chains = OligPool[:DP]
            del OligPool[:DP]
            chain = "-".join(chains)
            Polymers.append(chain)
    Polymers.extend(OligPool)
    #Saves and outputs the data
    filename3 = path+'Poly\\DP'+DPoli1+'-'+DPoli2+'_pU'+pUoli1+'-'+pUoli2+'_Poly'
    with open(filename3, 'wb') as out:
        pickle.dump(Polymers, out)

    return Polymers
```

Data Extraction. The raw simulation data, generated by the code described in the previous section, is saved as Python lists within .pkl files. Each list represents a specific polymer sample, and every line in that list corresponds to a single polymer chain.
The structure of the data is as follows:

- Each chain is a sequence of characters representing individual monomers or bonds.
- The following characters are used:
  "**g**": Represents the diol monomer.
  "**u**" and "**s**": Represent the two types of diacid monomers.
  "**-**": Represents the bond formed during "Phase 2" of the algorithm, linking two previously formed oligomers together to create the final polymer chain.

When analyzing a chain containing multiple combined oligomers, hyphens separate the original building blocks. A screenshot of an arbitrarily selected section of the raw **(U50)$_5$-alt-(U0)$_{10}$** copolymer data is provided below for reference.

```
's-gsgsg',
'u-gsgsgsgsgsgsgsg',
'gsgsgsgsgsgsgsg-u-g',
'gsgsgsgsgsgsg-u-gsgsgsgsgsgsgsgsgsgsgsgsgsgsgsgsg',
'gsgsgsgsgsg-sgsgugs-gsgsgsgsgsgsg',
'gsgsgsgsgsgsgsgsgsg-sgsgsgsgu-gsgsg',
'gsg-ugu-gsgsg',
'gsgsgsgsgsgsgsgsgsgsg-u-gsg',
'gsgsgsgsgsgsgsgsgsgsgsgsgsg-sgsgugsgs-gsgsgsgsgsg',
'gsgsg-u-g',
'gsgsgsg-ugsgugugsgugugugu-g',
'sgu-g-sgu-gsgsgsgsgsgsgsgsg',
'sgu-g-ugs-gsgsgsgsg',
'sgu-gsgsgsgsg-ugsgu-gsg',
'sgugugugugugugugugsgsgugugugugugs-gsgsgsgsg-ugugsgu-g',
'sgugs-gsgsgsgsgsg-ugugugsgugs-g',
'sgsgsgugu-g-sgugsgsgugu-gsgsgsg',
'sgsgu-g-ugugs-gsgsg',
```

Key Data Features:

- The hyphen separators always link a **g**-terminated diol oligomer with a **u** or **s**-terminated diacid oligomer.
- The oligomers within the chains have variable lengths. As discussed in the main text, the lengths within each initial oligomer pool follow a Flory distribution, and their overall **u**/**s** composition is determined by the inputs provided to the data generation function.
- Each polymer data file contains one million lines, representing one million individual polymer chains.

The python functions in the following section were used to extract the data presented in the main text.

Data Extraction Source Code

Note: The functions below require the raw data to be loaded from the ".pkl" files into the current Python environment. In all subsequent function definitions, the "data" argument refers specifically to this loaded polymer list. The returned outputs can be stored into temporary variables or saved.

Monomers per chain DP distribution (undigested)

Data used in Figure 8A and Figure 9A of the main text

```
from collections import Counter


def DPdistribution(data):
    #Creates an empty list to store data
    newdata = []
    #Iterates through each line in the dataset
    for chain in data:
        #Removes the hyphen separator between the oligomers
        #This hyphen is not a monomer and should not be included
        chain = chain.replace('-','')
        newdata.append(chain)
    #dist is a new list, but instead of the letters representing individual monomers,
    #each line becomes a single number, the length of the chain (monomers per chain)
    dist = [len(x) for x in newdata]
    #Counter() counts the frequency each chain length
    #and is converted to a dictionary
    #The keys are the DPs (# monomers per chain)
    #The values are the frequency with which that DP appeared in the data
    Distribution = dict(Counter(dist))
    #Distribution is the DP distribution
    return Distribution

```

Oligomers per chain distribution

Data used in Figure 8B of the main text

```
from collections import Counter


def OligsPerChain(data):
    #For every line in the data, Counter() counts the frequency that "-" appears
    #The keys are the number of "-" per line and the values are the
    #number of occurrences in the entire dataset

    #Every time two oligomers form a bond, the "-" separator is inserted
    #If a chain is composed of 5 oligomers, it will contain 4 "-"s
    temp = dict(Counter(s.count('-') for s in data))
    #To convert the distribution to the desired oligomers per chain, add 1 to the keys
    new_dict = {key + 1: value for key, value in temp.items()}
    #new_dict is the "Oligomers per chain" distribution
    return new_dict

```

Monomers per chain DP distribution (post-digestion)
Data used in Figure 9B in the main text.
Note that the initial data for Figure 9D and Figure 11 were generated with this function, but conversion to “repeat units per chain” as described in Figure 9C and the main text was performed separately in excel.

```
from collections import Counter

def digestionDP(data):
    #The distribution generator using Counter() is the same
    #way it was used in OligsPerChain() and DPdistribution()
    #But now we digest (split) the polymers into fragments anytime a "u" appears

    #empty list to store new data
    digested = []
    for chain in data:
        #removes the hyphen separator, "-" is not a monomer
        chain = chain.replace('-','')
        #each line is searched, and anytime a "u" appears,
        #the line is split into fragments
        fragments = chain.split('u')
        #The fragments are added to the empty list above
        digested.extend(fragments)
    #generates the length distribution of the fragments
    dist = [len(x) for x in digested]
    temp = dict(sorted(Counter(dist).items()))

    #temp is the post-digestion DP distribution
    return temp
```

Post-digestion $\overline{DP}$, $\overline{DP}_{\ell}$, $Đ_{DP}$ calculations (Eq 4-6 of the main text)

Data used in Table 2 of the main text

```
from collections import Counter
from decimal import Decimal

def AvgDPs(data):
    digested = []
    RUcount = []
    #remove hyphens and digest the polymers
    for chain in data:
        chain = chain.replace('-','')
        fragments = chain.split('u')
        digested.extend(fragments)
    #count the number of monomers per chain. Each monomer is assigned
    #a value of 0.5 so the results are with respect to number of repeat units
    for fragment in digested:
        count = 0
        for monomer in fragment:
            if monomer == 's':
                count += 0.5
            elif monomer == 'g':
                count += 0.5
            else:
                pass
        #append the count to the list RUcount
        RUcount.append(count)
    #This gives the length distribution of the post-digestion data
    #with respect to repeat units per chain. Increments of 0.5 are allowed.
    Distribution = dict(Counter(RUcount))

    #creates a list of the DPs and the relative abundances separately
    #to be used in calculations of avg DP and DP dispersity
    DP = list(Distribution.keys())
    Num = list(Distribution.values())

    #creates two new lists, each line in "products" is DPi * Ni
    #Each line in "products2" is DPi^2 * Ni
    total = sum(Num)
    products = []
    products2 = []
    for num1, num2 in zip(DP, Num):
        products.append(num1 * num2)
        products2.append(num1 **2 * num2)
    #calculations of Avg DP, Length weighted avg DP and DP dispersity
    mons = sum(products)
    chains = round(Decimal(total),2)
    avgDP = round(Decimal(mons/total),2)
    avgDPw = round(Decimal(sum(products2)/sum(products)),2)
    DDP = round(Decimal(avgDPw/avgDP),2)
    #returns values for the post-digestion polymer
    #mons = total number of monomers in the entire dataset
    #chains = total number of chain fragmentsin the entire dataset
    #avgDP = the average DP (Repeat units per chain)
    #avgDPw = the length weighted average DP (Repeat units per chain)
    #DDP = DP dispersity
    return str(mons), str(chains), str(avgDP), str(avgDPw), str(DDP)
```

In evaluating the GS block-length distributions presented in Figure 10 of the main text, the discussion of **$(U53)_5$-*alt*-$(U0)_{11}$** (derived from discrete oligomers) establishes that:

"… short blocks, e.g., **$(GS)_0$** or **$(GS)_1$**, and longer blocks, e.g., **$(GS)_5$**, arise with relatively high probability, while certain intermediate lengths are impossible, e.g., **$(GS)_3$** or **$(GS)_4$**."

To elaborate on this further, every DP 11 diol (0% U) contains 5 full repeat units. The DP 5 diacid (53% U) can contain anywhere from 0-2 complete repeat units. After "dimerization", GS block-lengths of 6-7 can emerge, but lengths of 3-4 GS repeat units are not possible. Incorporating polydisperse oligomers relaxes these constraints, making intermediate block-lengths possible, albeit with low statistical probability. Figure S23 includes a table of possible sequences for the DP 5 and DP 11 oligomers, as well as some of the DP 16 "dimers".

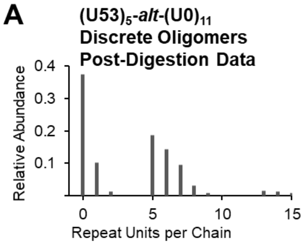

B **Possible Sequences**

**Discrete Oligomers** Polymer Sequence DP 5 53% U	DP 11 0% U	Fragment Length Number of Repeat Units
SGUGU		0
SGUGS		
UGSGU		1
UGSGS		
SGSGS		2
	Not possible	3
	Not possible	4
	GSGSGSGSGSG	5
SGUGS	- GSGSGSGSGSG	6
UGSGS	- GSGSGSGSGSG	7

Figure S23. A) Post-digestion distribution for the P-S copolymer **$(U53)_5$-alt-$(U0)_{11}$** made from discrete oligomers. B) Table of possible sequences and GS block-lengths for the DP 5 diacid monomer, the DP 11 diol monomer and the DP 16 dimer. Because the DP 11 diol has no **U** monomer, it must have a **GS** block length of 5. The DP 5 diacid can have a max **GS** block length of 2. The number of repeat units is consistent with the definition set forth in the main text, as described in Figure 9.
